# Chronological and biological aging of the human left ventricular myocardium: Analysis of microRNAs contribution

**DOI:** 10.1111/acel.13383

**Published:** 2021-06-06

**Authors:** Estel Ramos‐Marquès, Laura García‐Mendívil, María Pérez‐Zabalza, Hazel Santander‐Badules, Sabarathinam Srinivasan, Juan Carlos Oliveros, Rafael Torres‐Pérez, Alberto Cebollada, José María Vallejo‐Gil, Pedro Carlos Fresneda‐Roldán, Javier Fañanás‐Mastral, Manuel Vázquez‐Sancho, Marta Matamala‐Adell, Juan Fernando Sorribas‐Berjón, Javier André Bellido‑Morales, Francisco Javier Mancebón‑Sierra, Alexánder Sebastián Vaca‑Núñez, Carlos Ballester‐Cuenca, Manuel Jiménez‐Navarro, José Manuel Villaescusa, Elisa Garrido‐Huéscar, Margarita Segovia‐Roldán, Aida Oliván‐Viguera, Carlos Gómez‐González, Gorka Muñiz, Emiliano Diez, Laura Ordovás, Esther Pueyo

**Affiliations:** ^1^ Biomedical Signal Interpretation and Computational Simulation group (BSICoS) Aragón Institute of Engineering Research University of Zaragoza Zaragoza Spain; ^2^ BSICoS IIS Aragón Zaragoza Spain; ^3^ Bioinformatics for Genomics and Proteomics National Center of Biotechnology‐ Spanish National Research Council Madrid Spain; ^4^ Biocomputation unit IACS Zaragoza Spain; ^5^ Department of Cardiovascular Surgery University Hospital Miguel Servet Zaragoza Spain; ^6^ Heart Area Hospital Clínico Universitario Virgen de la Victoria, CIBERCV IBIMA, Universidad de Málaga, UMA Málaga Spain; ^7^ UGC Heart Area Cardiovascular Surgery Department Hospital Universitario Virgen de la Victoria de Málaga Fundación Pública Andaluza para la Investigación de Málaga en Biomedicina y Salud (FIMABIS) CIBERCV Enfermedades Cardiovasculares Instituto de Salud Carlos III University of Málaga Madrid Spain; ^8^ Department of Pathology San Jorge Hospital Huesca Spain; ^9^ Institute of Experimental Medicine and Biology of Cuyo (IMBECU) CONICET Mendoza Argentina; ^10^ ARAID Foundation Zaragoza Spain; ^11^ Biomedical Research Networking Center in Bioengineering Biomaterials and Nanomedicine (CIBER‐BBN) Zaragoza Spain

**Keywords:** biological aging, biomarkers, gene regulation network, heart aging, microRNA, transcriptomic age marker

## Abstract

Aging is the main risk factor for cardiovascular diseases. In humans, cardiac aging remains poorly characterized. Most studies are based on chronological age (CA) and disregard biological age (BA), the actual physiological age (result of the aging rate on the organ structure and function), thus yielding potentially imperfect outcomes. Deciphering the molecular basis of ventricular aging, especially by BA, could lead to major progresses in cardiac research. We aim to describe the transcriptome dynamics of the aging left ventricle (LV) in humans according to both CA and BA and characterize the contribution of microRNAs, key transcriptional regulators. BA is measured using two CA‐associated transcriptional markers: *CDKN2A* expression, a cell senescence marker, and apparent age (AppAge), a highly complex transcriptional index. Bioinformatics analysis of 132 LV samples shows that *CDKN2A* expression and AppAge represent transcriptomic changes better than CA. Both BA markers are biologically validated in relation to an aging phenotype associated with heart dysfunction, the amount of cardiac fibrosis. BA‐based analyses uncover depleted cardiac‐specific processes, among other relevant functions, that are undetected by CA. Twenty BA‐related microRNAs are identified, and two of them highly heart‐enriched that are present in plasma. We describe a microRNA‐gene regulatory network related to cardiac processes that are partially validated *in vitro* and in LV samples from living donors. We prove the higher sensitivity of BA over CA to explain transcriptomic changes in the aging myocardium and report novel molecular insights into human LV biological aging. Our results can find application in future therapeutic and biomarker research.

## Graphical abstract

The biological age, measured by transcriptional markers, explains better than the chronological age the transcriptional dynamics of the human aging myocardium. microRNAs that change expression in the aging left ventricle are identified. Such microRNAs potentially regulate genes with cardiac‐specific functions. Cardiac‐enriched microRNAs that increase or decrease with biological age are present in plasma. They are thus candidate biomarkers to represent the biological age of the heart.

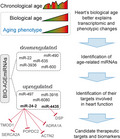

## 1 INTRODUCTION

Age is the main risk factor for heart failure and other cardiovascular diseases. Aged hearts undergo structural and functional changes at multiple levels that contribute to the pathogenesis of the disease. At the molecular level, these changes are far from being fully characterized, particularly in humans (Obas & Vasan, 2018). On the one hand, studies carried out mainly in animal models (of considerably reduced lifespan, homogeneous genetic backgrounds and housing in controlled environments) limit translation to humans. On the other hand, the difficulties in discerning between the effects of aging and disease in humans, in conducting longitudinal studies and in obtaining cardiac samples, all contribute to this lack of knowledge.

Studies investigating human aging are mainly conducted transversally in relation to chronological age (CA). But, CA is a mere time index that does not necessarily explain the biological condition of an individual or an organ. The biological age (BA) of an organ, perceived as its actual structural and functional state, is a composite of both genetic and environmental factors acting together over time. The significance of BA on top of CA in cardiovascular disease has been studied. BA has been associated with improved prognosis capacity after ischemic stroke as compared to CA (Soriano‐Tárraga et al., 2018) and with higher risk of cardiovascular disease (Lind et al., 2018). However, the transcriptional dynamics of the aging heart, and specifically the left ventricle (LV), have not been investigated yet from a biological point of view.

Biological age is a complex parameter that can be scored by different approximations such as clinical markers, functional tests, or molecular indicators. Cellular senescence is a fundamental mechanism of aging tightly related to the expression of the cell‐cycle regulator and cell senescence marker *P16* (expressed from the *CDKN2A* locus) (Krishnamurthy et al., 2004). *P16*/*CDKN2A* is considered a BA marker in human skin and peripheral blood cells (Liu et al., 2009; Waaijer et al., 2012). The number of P16/CDKN2A^+^ senescent cells increases with age in various human tissues (Idda et al., 2020) including the heart (Chimenti et al., 2003). In the mouse heart, the pool of p16/Cdkn2a^+^ senescent cells also increases with age (Grosse et al., 2020; Torella et al., 2004) and their clearance reverts age‐related phenotypes in cardiovascular disorders and improves heart function in chronologically aged mice (Shimizu & Minamino, 2019; Walaszczyk et al., 2019; Zhu et al., 2015). Yet, to the best of our knowledge, *CDKN2A* expression has not been distinctly used as a cardiac BA marker. Other highly complex transcriptomic indices could as well account for the aging rate variability of an organ. Apparent age (AppAge) is an unbiased BA index computed from age‐related transcriptional indicators (Rhinn & Abeliovich, 2017) that adjusts the CA of an individual by his/her aging rate.

microRNAs (miRNAs) are relevant regulators of biological processes that inhibit expression of their target genes (De Lucia et al., 2017). miRNAs seem to have a role in the regulation of heart aging. For example, miR‐22, miR‐18, miR‐19a, and miR‐19b, and miR‐17‐3p have been associated with senescence, apoptosis, autophagy, hypertrophy, and fibrosis in the heart, mainly in animal models (Gupta et al., 2016; Jazbutyte et al., 2013) and miR‐34 is a master regulator of cardiac aging in mice with age‐related expression in human right atrium (Boon et al., 2013). Little is known, though, about their role in humans. Circulating miRNAs have been described as biomarkers of aging and frailty (Rusanova et al., 2018), but since not all organs seem to age at the same rate (Yang et al., 2015), organ‐specific circulating biomarkers should be used to estimate heart aging.

This study aims at describing gene and miRNA expression changes in the human LV induced by CA and transcriptomic BA through transcriptomic analysis of LV samples of the extensive GTEx RNA‐seq dataset. We assess the potential of simple (*CDKN2A* expression) and highly complex (AppAge) transcriptomic age (TA) markers to uncover cardiac‐specific age‐related processes, and we demonstrate TA markers relation to an aging phenotype. Also, our study deciphers the potential contribution of miRNAs in modulating age‐related transcriptional changes and establishes and partially validates a downstream regulation network of genes involved in the myocardial function. Finally, cardiac‐enriched BA‐regulated miRNAs are identified and detected in plasma, proposing them as potential secreted biomarkers of the aging myocardium. This work sets the ground for future studies addressing the development of LV‐specific miRNA‐based anti‐aging therapies and the assessment of cardiac biomarkers to stratify the risk for BA‐related heart diseases.

## 2 METHODS

A detailed description of the methods is provided in the Supporting Information.

### 2.1 Donors and sample selection

RNA‐seq data of LV tissue were obtained from 132 males (20 to 70 y.o.) of the GTEx study Version 7 (Carithers et al., 2015) whose reported cause of death was not related to cardiovascular diseases (non‐cardiac deaths, NCD) (Table S1).

### 2.2 Hierarchical clustering and age sample distribution analysis

Normalized expression values (Table S2) were obtained from raw expression (read counts) of RNA‐seq samples.

AppAge of each individual was calculated in this study cohort, from 2863 age‐related genes (Figure S1) according to methods described in Rhinn and Abeliovich (2017). For details, see supplement.

Samples were hierarchically clustered by normalized expression values of the whole transcriptome. Histograms and kernel smoothing function fits were calculated for each of the two main groups of the dendrogram (defined by the two earliest separated branches), according to three aging variables (Table S3): AppAge, *CDKN2A* expression, and CA.

### 2.3 Quantification of myocardial fibrosis

Hematoxylin‐eosin histology images corresponding to each of the 132 NCD donors were obtained from the GTEx database. The amount of cardiac fibrosis was automatically quantified using custom‐written software.

### 2.4 Whole transcriptome gene set enrichment analysis

Functional groups of genes enriched or depleted in old individuals according to each of the age markers were analyzed by GSEA (Table S4).

### 2.5 Identification of age‐related miRNAs

BIO‐AGEmiRNAs were identified from all miRNAs in the RNA‐seq analysis as those differentially expressed in old versus young according to *CDKN2A* expression values and additionally showing significant Spearman's correlation with *CDKN2A* expression. Further details can be found in the supplementary methods (Tables [Link], [Link]).

### 2.6 Selection of mirror targets for age‐related miRNAs

For each BIO‐AGEmiRNA, predicted target genes were retrieved from miRWalk2.0 database (Dweep et al., 2011). To refine the sensitivity in putative target identification and to find genes mainly regulated by each BIO‐AGEmiRNA, the predicted targets were filtered to mirror the expression profile of their associated BIO‐AGEmiRNA (Table S7).

### 2.7 Gene regulation network by age‐related miRNAs

A network was constructed with BIO‐AGEmiRNAs, and their associated mirror targets annotated into cardiac‐related gene ontology groups (Table S8).

### 2.8 Luciferase reporter assay

Cardiac targets were cloned in the reporter vector (pmirGLO‐P2A‐3FLAG‐MCS) downstream of *Firefly* luciferase (Table S9) and co‐transfected in the presence/absence of miRNA mimics (Riboxx) in Hek293 cells. Luciferase assays were done with Dual Glo (Promega) according to manufacturer's instructions.

### 2.9 Tissue specificity assessment

LV expression specificity of each of the BIO‐AGEmiRNAs was determined by calculating the Specificity Measure SPM (Pan et al., 2013) and by determining significant differences in BIO‐AGEmiRNA expression levels across tissues in pairwise comparisons.

### 2.10 Statistical analysis

SPSS version 22 was used for statistical analyses. Spearman's correlation analysis was used to test the strength and direction of association between two variables. Mann–Whitney test was used to assess differences between two independent groups. The significance threshold was established at *p* = 0.05 for Mann–Whitney test and FDR = 0.05 for the rest of analyses.

## 3 RESULTS

### 3.1 *CDKN2A* expression and AppAge represent LV transcriptomic age

We first aimed to understand whether age was embodied in the transcriptome to subsequently compare the capacity of CA and TA (*CDKN2A* expression, AppAge) markers to represent gene expression differences with age in the 132 male individuals of the GTEx consortium with NCD cause of death.

**FIGURE 1:**
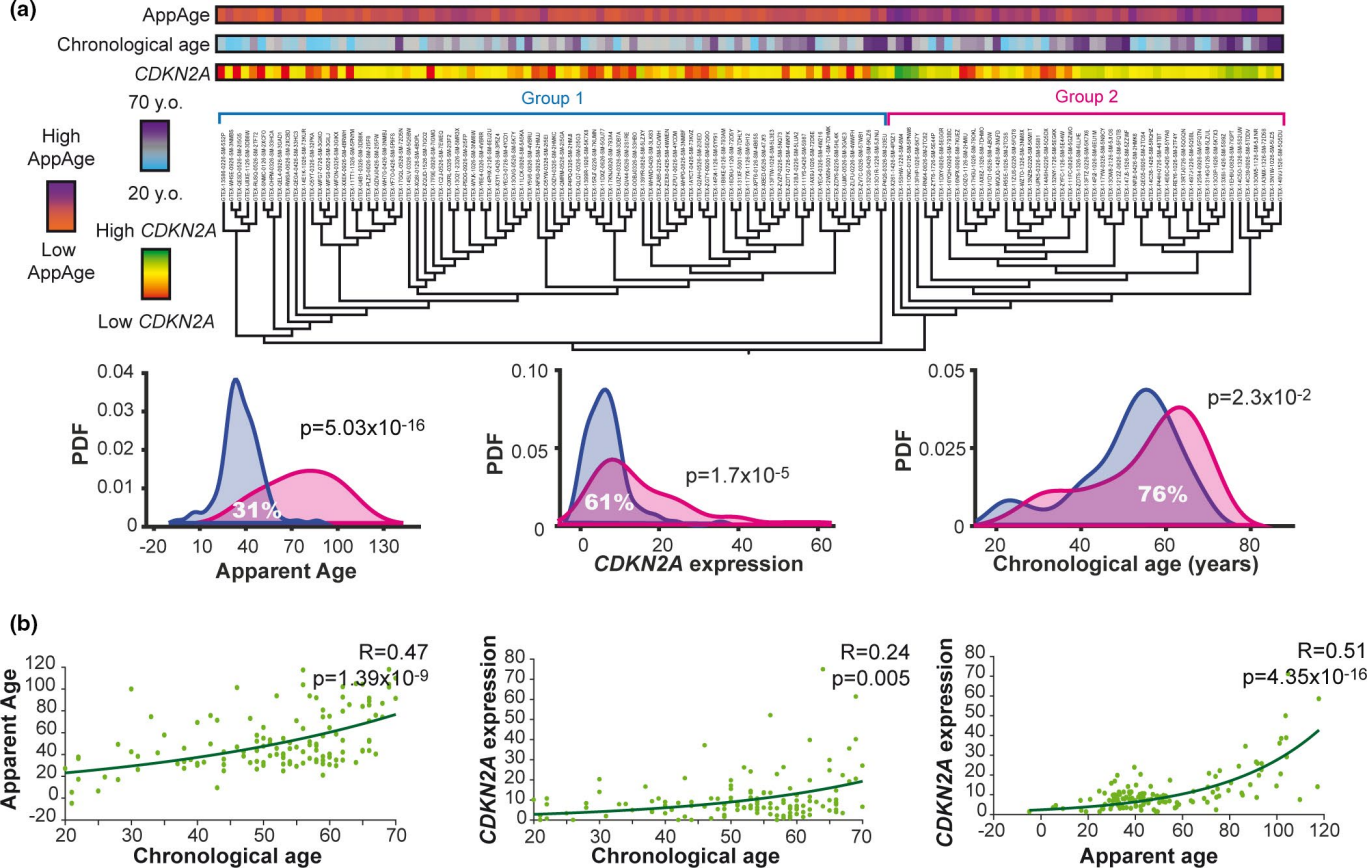
Relationship between human LV transcriptome and aging. (a) Dendrogram representing hierarchical clustering classification of individuals based on the whole transcriptome analysis. Groups 1 (blue) and 2 (pink) are determined by early separated branches. Color bars on top of the dendrogram represent CA, AppAge, or *CDKN2A* expression of each individual according to the indicated color scales on the left. Below, the histograms show the distributions of CA, AppAge, or *CDKN2A* expression levels for the two groups of the dendrogram. Y‐axes show the probability density function (PDF). Percentages of overlap and *p*‐values (Mann–Whitney test) are shown. (b) Correlation between aging markers (CA, AppAge, and *CDKN2A* expression). Dots indicate individual data, while the line represents the fitted function. Correlation coefficient HAS and *p*‐values (Spearman) are shown

Hierarchical clustering using the whole transcriptome categorized individuals into two main groups (Figure 1a). Group 1 (83 individuals) contained a larger proportion of individuals with low CA, AppAge, and *CDKN2A* expression, while group 2 (49 individuals) had a larger number of individuals with high CA, AppAge, and *CDKN2A* expression values. Sample distributions according to the age markers were significantly different between group 1 and group 2, with the overlapping area between the two groups being 31% for AppAge, 61% for *CDKN2A* expression, and 76% for CA (Figure 1a). These results indicated that aging was represented in the whole LV transcriptome and that AppAge, a highly complex TA marker, better aligned with the hierarchical clustering classification followed by *CDKN2A* expression and CA.

We further assessed the biological significance of the TA markers by correlation analysis with CA. AppAge and *CDKN2A* presented significant correlation with CA, with Spearman R coefficients being 0.47 and 0.24, respectively (Figure 1b). Interestingly, although they were independently calculated and relied on a largely different number of genes (1 vs. 2863), a clear correlation was found between the two TA markers, AppAge and *CDKN2A*, with a Spearman R coefficient of 0.51 (Figure 1b).

Thus, the TA markers AppAge and *CDKN2A* are correlated and represent the transcriptomic characteristics of the analyzed population better than CA for the human LV.

### 3.2 TA markers are associated with a cardiac aging phenotype linked to heart dysfunction

**FIGURE 2:**
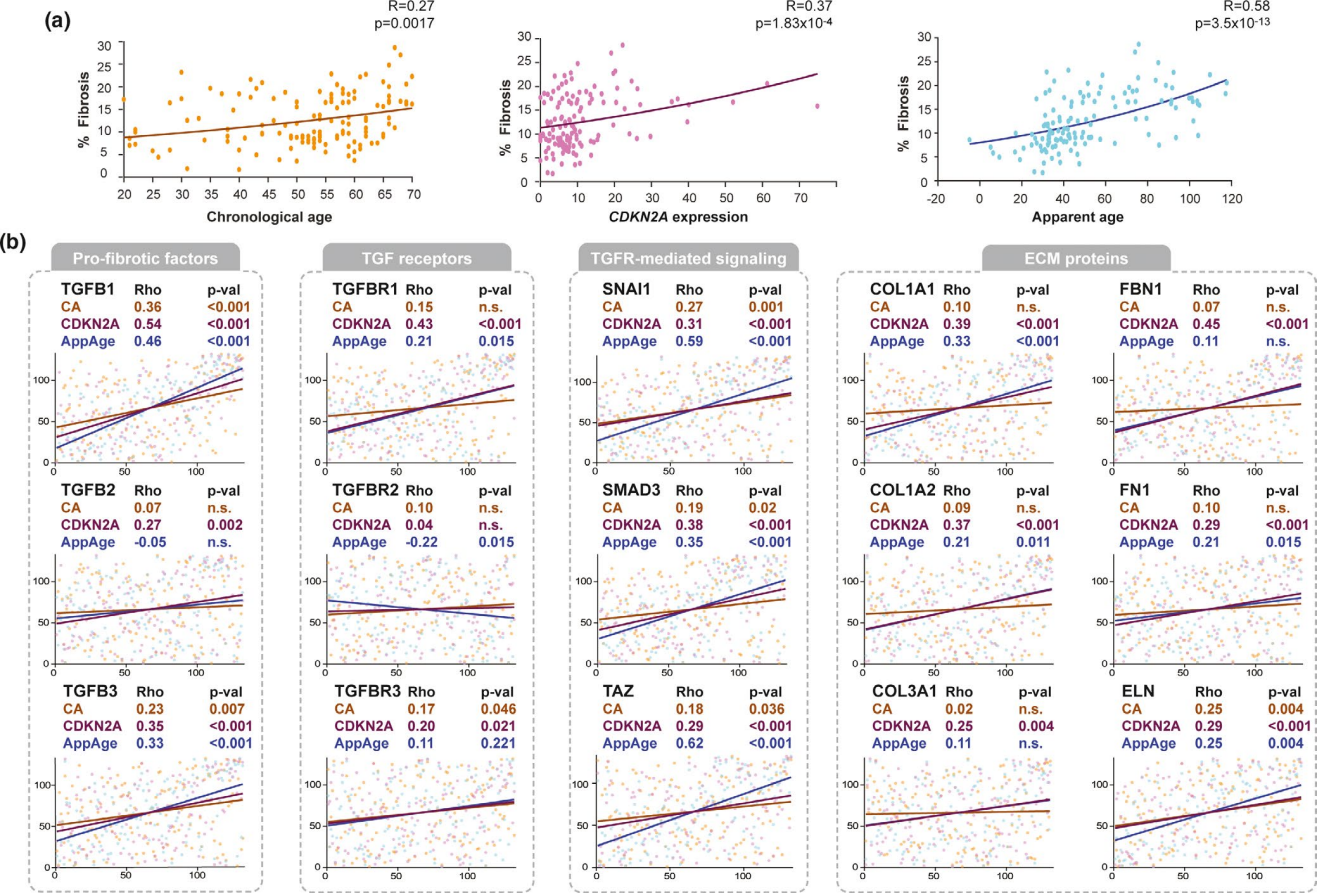
Correlations between aging markers and fibrosis. (a) Correlation between percentage of interstitial fibrosis and age markers (CA, *CDKN2A*, and AppAge). Dots indicate individual data, while the line represents the fitted function. Correlation coefficient HAS and p‐values (Spearman) are shown. (b) Correlation between fibrosis‐related genes and age markers (CA, *CDKN2A*, and AppAge). Correlation coefficient HAS and *p*‐values (Spearman) are shown (*p < 0.05; **p < 0.01; ***p < 0.001). For ease of understanding, axes show the 132 samples ordered by the three age markers (X axis) and each gene expression level (Y axis)

To provide biological validation for the age markers, we investigated their relation with myocardial fibrosis, a feature of structural remodeling of the aging heart that is related to impaired function (Gazoti Debessa et al., 2001; Piek et al., 2016). The amount of fibrosis found in the LV was significantly correlated with all age markers with Spearman R being 0.27, 0.32, and 0.58 for CA, *CDKN2A* expression, and AppAge, respectively (Figure 2a, Table S10). Looking closer at the mechanism, the expression of genes involved in the pro‐fibrotic TGF‐β pathway showed that most of the TGF‐β factors, receptors, and downstream signaling molecules, were associated with all three aging markers (Figure 2b). Instead, genes encoding extracellular matrix (ECM) proteins, final effectors of the fibrotic phenotype, were all significantly associated with both TA markers, but not with CA (except for Elastin, *ELN*) (Figure 2b). This is in line with the results exposed above, where CA shows the weakest correlation to the percentage of fibrosis.

Altogether, the results show that both TA markers, AppAge and *CDKN2A* expression, are not only good descriptors of transcriptomic age but also explain, better than CA, the age‐related fibrotic phenotype and its main mechanisms at the single‐gene level.

### 3.3 Biological and chronological age‐based analysis reveals differentially altered functions in the aged human LV

Next, we examined the potential of the three age markers to uncover age‐related genes by differential gene expression analysis. Samples were classified in CA decades (20–30, 31–40, 41–50, 51–60, and 61–70 y.o.) or fifths according to *CDKN2A* expression and AppAge (P20/40/60/80/100). CA identified 657 differentially expressed genes (DEGs) between young (20–30 y.o.) and old individuals (61–70 y.o.), substantially much less than the 14,437 and 15,670 discovered by *CDKN2A*‐ and AppAge‐based analysis comparing young (P20) and old (P100) BA individuals, respectively. More than 70% of the CA‐upregulated or downregulated genes in old as compared to young donors were also retrieved by *CDKN2A* and AppAge, once again confirming their link to age (Figure S2). Moreover, there was more than 93% overlap of DEGs between TA markers.

**FIGURE 3:**
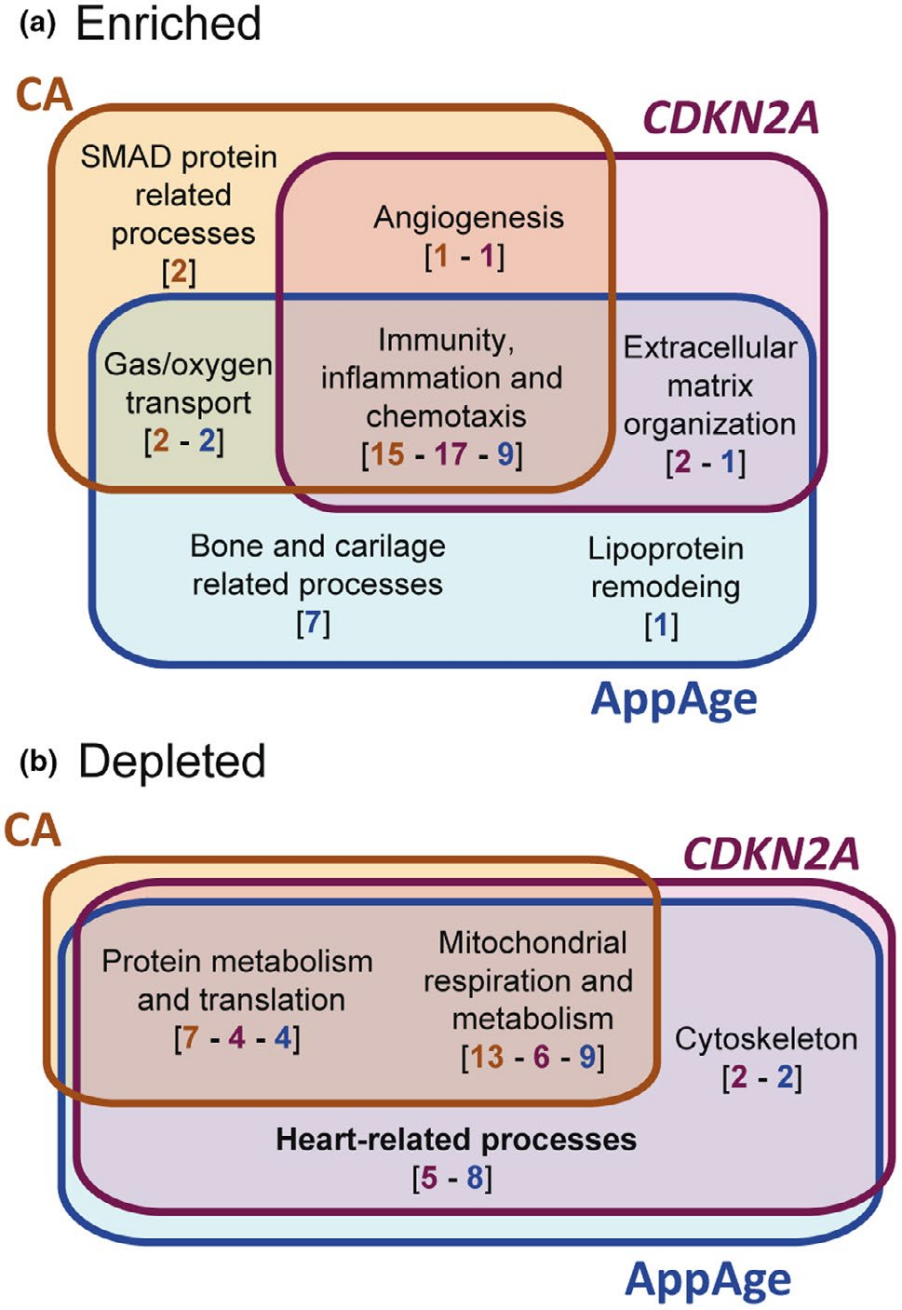
Analysis of enriched and depleted functions in biologically and chronologically old individuals. (a) Enriched functions in CA‐, AppAge‐, or *CDKN2A*‐classified samples, and the overlapping between them. Colored numbers in brackets indicate the number of Gos gathered into the broader biological functions in each age‐based analysis (orange, blue, and purple for CA, CDKN2A, and AppAge, respectively). (b) Depleted functions in CA‐, AppAge‐, or CDKN2A‐classified samples, and the overlapping between them. Broad functions and number of groups, follow the same representation as in (a)

These findings led us to evaluate enrichment or depletion of functional processes in old individuals using gene set enrichment analysis (GSEA) (Table S11). A total of 64, 895 and 1,684 gene ontology groups (GOs) were significantly enriched by CA, *CDKN2A*, or AppAge, respectively. The top 20 depleted and enriched GOs in old versus young (Table S12) were classified into wider categories to understand which general functions were altered according to the three age markers. In the CA‐, AppAge‐, and *CDKN2A*‐classified samples, functions related to immunity, inflammation, and chemotaxis were enriched (Figure 3a), while those related to mitochondrial respiration, metabolism, and protein metabolism and translation were depleted (Figure 3b). These functional groups belong to the aging landmarks “inflammation,” “mitochondrial dysfunction,” and “loss of protein homeostasis” (López‐Otín et al., 2013).

A functional group enriched in high CA‐ and high *CDKN2A*‐classified samples was related to angiogenesis. Also, groups related to gas and oxygen transport, in line with the angiogenic process, were enriched both in high CA‐ and high AppAge‐classified samples. Groups related to ECM organization emerged in high *CDKN2A* and high AppAge donors only, while SMAD processes appeared enriched in CA‐old individuals only. Another substantial difference between all the age markers was the identification by only high AppAge of 7 enriched GOs involved in bone and cartilage processes.

Of note, despite *CDKN2A* being a cell‐cycle regulator, only two enriched groups out of 795 were related to cell cycle‐related processes (“Regulation of cell‐cycle arrest” and “Negative regulation of cell‐cycle arrest”), but they had low normalized enrichment score (Table S11).

Depleted functional groups related to heart function showed up in high‐AppAge‐ (8 GOs) and high‐*CDKN2A*‐ (5 GOs) classified samples. These included processes such as “Heart process,” “Cardiac muscle contraction,” “Cardiac muscle cell action potential,” or “Cell communication involved in cardiac conduction” (Table S12). Also, GOs related to cytoskeleton processes (“Regulation of actin filament‐based movement,” “Myofibril assembly” or “Actin‐mediated cell contraction”) were present in the TA‐based analysis. None of these heart‐ or cytoskeleton‐related processes were retrieved in CA‐old individuals.

Altogether, both TA markers demonstrated to represent age in the human LV transcriptome (Figure 1), proved association with an aging phenotype (Figure 2), and unveiled depleted heart‐related processes (Figure 3) with overall higher sensitivity than CA, being AppAge more precise and containing most of the information retrieved by *CDKN2A*. The TA markers significantly correlated among them, but the use of AppAge has relevant drawbacks. Its high complexity (computed from 2863 genes in this data set) would preclude the validation of RNA‐seq data on new LV specimens at the single‐gene level. In addition, AppAge computation is cohort‐dependent, what would prevent comparison between studies. Therefore, AppAge allowed us to confirm the findings observed for *CDKN2A* in the GTEx NCD cohort, but, for our subsequent analysis, BA is estimated with the TA marker *CDKN2A* expression.

### 3.4 miRNAs are involved in biological aging of the human LV

We next investigated whether miRNAs could contribute to the transcriptomic regulation of cardiac functions altered with BA.

BA‐related miRNAs, BIO‐AGEmiRNAs, were identified as those differentially expressed and presenting significant correlation with *CDKN2A* expression. In total, 20 BIO‐AGEmiRNAs were identified, 14 were upregulated and 6 downregulated (Table 1).

**TABLE 1:** List of up/down‐regulated BIO‐AGEmiRNAs in elder versus young individuals

BIO‐AGEmiRNA	
Up‐regulated	Down‐regulated
MIR4435‐1HG	MIR600HG
hsa‐mir‐6080	hsa‐mir‐490
MIR24‐2	MIR3936
MIR497HG	MIR635
MIR3916	MIR22HG
MIR503HG	MIR17HG
MIR3911	
MIR155HG	
MIR1304	
MIR3648	
MIR296	
MIR210HG	
hsa‐mir‐7162	
MIR4461	

Subsequently, a total of 18,679 putative targets of BIO‐AGEmiRNAs were found in the miRWalk database (Sticht et al., 2018). Of these, only 10,561 fitted mirror expression pattern to their corresponding regulating miRNAs (Figure S3). Additional filters based on differential expression between BA‐young and old groups and significant correlation with *CDKN2A* expression led to the final number of 5,879 mirror targets: 3,658 from upregulated and 2,221 from downregulated BIO‐AGEmiRNAs.

In conclusion, our approach identifies a number of BIO‐AGEmiRNAs and their mirror targets with a clear BA‐related profile.

### 3.5 Biological (*CDKN2A*) age‐related miRNAs are predicted to orchestrate heart‐related processes during aging

**FIGURE 4:**
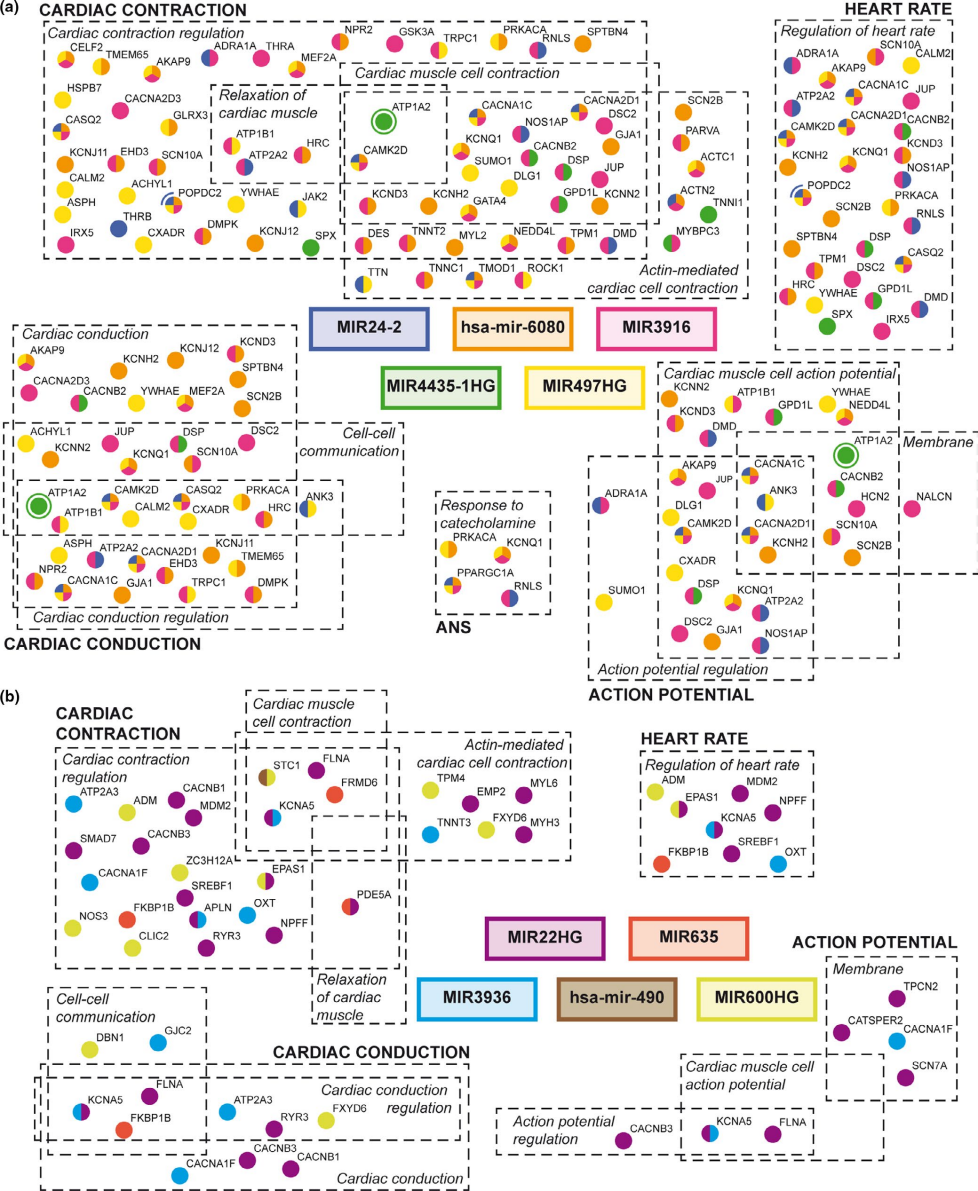
Predicted BIO‐AGEmiRNAs gene regulatory networks of cardiac‐related functions. Network for the top 5 upregulated (a) or downregulated (b) BIO‐AGEmiRNAs. Mirror targets are shown as color‐coded dots according to their putative regulating miRNA. Sections of cardiac Gos are delimited by dashed‐lines and functional categories are shown in capital letters

To understand how LV function along aging could be modulated by miRNAs, a downstream gene regulation network of heart‐related functions was established. Mirror targets belonging to cardiac GOs (Table S13) were selected and represented in five functional categories. Due to space limitations, only the top 5 induced and repressed BIO‐AGEmiRNAs were represented in the networks (Figure 4).

The top 5 upregulated BIO‐AGEmiRNAs had 3,524 mirror targets and 72 of them belonged to cardiac GOs (Figure 4a). *MIR3916* had the largest contribution in the network potentially regulating 63% of the cardiac genes all over the functional categories. Although the majority of cardiac mirror targets were linked to one or two BIO‐AGEmiRNAs, some genes such as *CAMK2D*, *CACNA2D1*, *CACNA1C*, or *TMOD1* were modulated by 4 out of the 5 BIO‐AGEmiRNAs.

Out of the 2,213 mirror targets of downregulated BIO‐AGEmiRNAs, 34 of them were annotated into cardiac GOs. *MIR22HG* had the largest contribution to the network (Figure 4b), regulating 52% of the genes. In this case too, most of these genes were linked to only one BIO‐AGEmiRNA.

**FIGURE 5:**
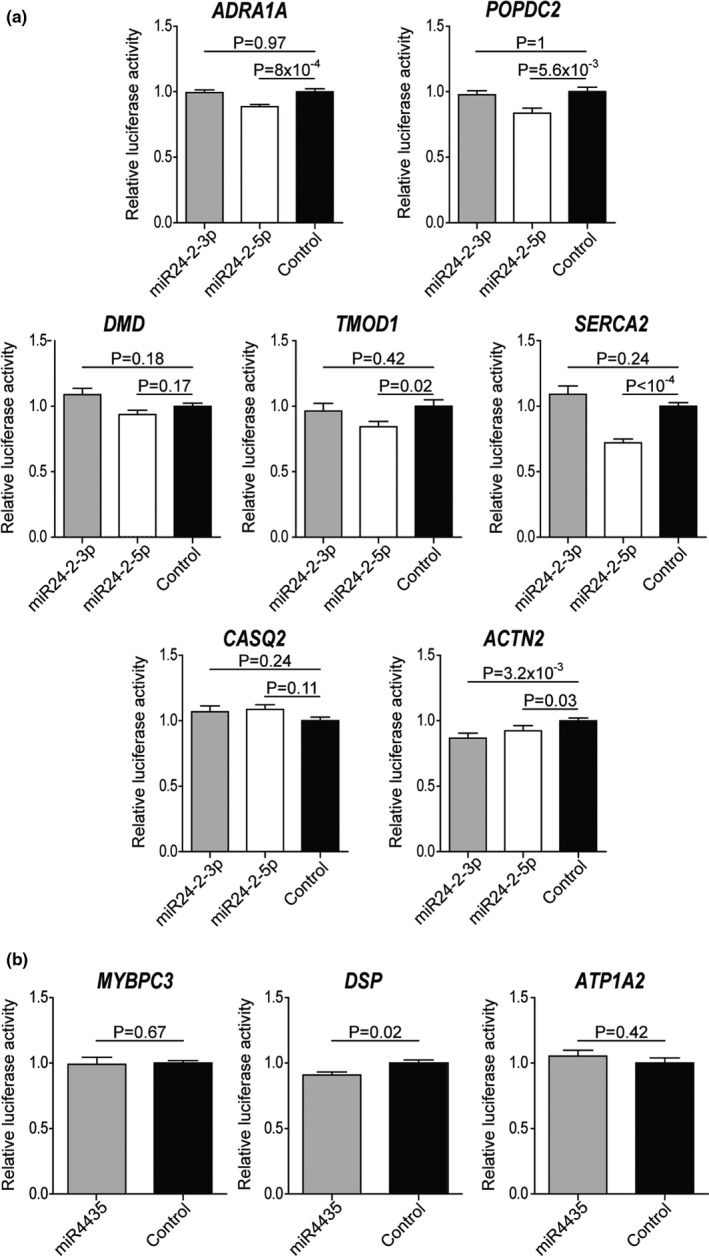
BIO‐AGEmiRNAs and cardiac target interactions. Luciferase expression assay performed for miR24‐2‐3p or miR24‐2‐5p (a) and miR4435 (b). Cardiac target under study is shown over each bar chart. Y‐axes show the detected luciferase signal, normalized to the control experimental condition (non‐targeting miRNA). Error bars show SEM and statistical analysis by Mann–Whitney test

To validate the bioinformatics approach used to construct the network, we assessed *in vitro* some of the predicted interactions. Two BIO‐AGEmiRNAs (hsa‐miR‐24–2 and hsa‐miR‐4435) and ten of their cardiac targets were chosen based on their highest correlation coefficient with *CDKN2A* expression (Figure 5). Our results showed that hsa‐mir‐24‐2‐5p significantly interacted with *ADRA1A*, *POPDC2*, *SERCA2*, *TMOD*, and *ACTN2* gene sequences as compared to a non‐targeting miRNA control. *ACTN2* also showed positive interaction with hsa‐miR‐24‐2‐3p, but *CASQ2* and *DMD* did not respond to the treatment with any branch of hsa‐miR‐24‐2 mimics. As per hsa‐miR‐4435, only the vector containing the *DSP* target sequence yielded interaction. In total, 6 out of 10 tested interactions proved positive indicating the high‐predictable value of the network.

Because the bioinformatics analysis in the GTEx population could be affected by donor and/or sample covariates (i.e., cause of death, RIN, postmortem interval, premortem conditions), we preliminarily analyzed gene expression in a small group of LV samples from living donors, ideally devoid of this possible source of variability. As compared to GTEx donors, the trend of change in the relative expression median values was conserved for most of the analyzed genes with increasing BA (Figure [Link], [Link], Tables [Link], [Link]).

In summary, we identified miRNAs upregulated and downregulated with BA and their putative regulated targets. According to our analysis, BA‐related miRNAs might play a pivotal role regulating genes involved in human LV function. This study establishes the first bioinformatic BIO‐AGEmiRNA gene regulation network of LV dysfunction associated with age in humans.

### 3.6 Identification of LV‐enriched biomarkers of aging

**FIGURE 6:**
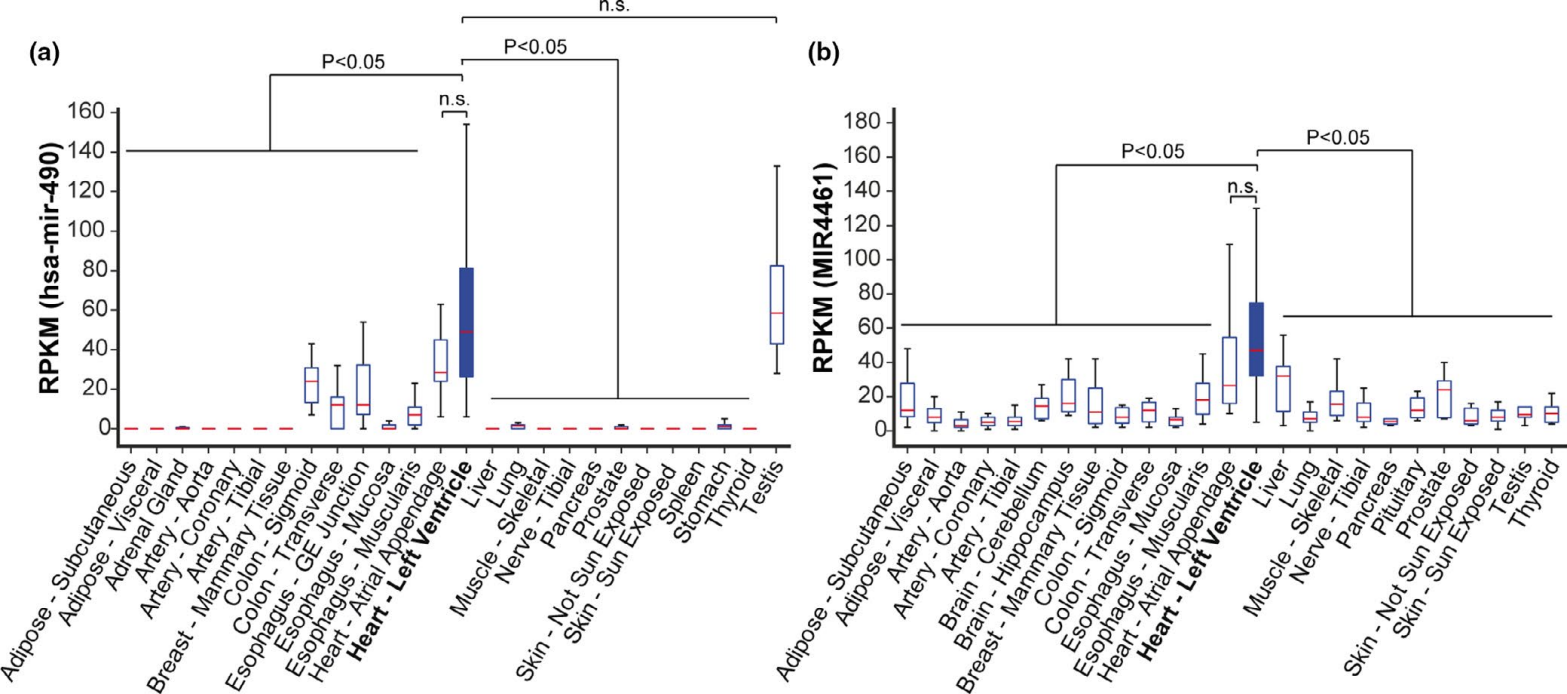
Assessment of BIO‐AGEmiRNA tissue specificity. Box plots showing the expression levels (RPKM) of the two BIO‐AGEmiRNAs with the highest LV specificity: MIR4461 (a) and has‐mir‐490 (b). Median RPKM values are shown in red lines and p‐values from Mann–Whitney tests comparing LV with each of the other tissues are indicated

Finally, we sought to identify age‐related LV‐enriched miRNAs that could eventually be used to assess cardiac BA in peripheral body fluids. We assessed BIO‐AGEmiRNAs expression levels in different human tissues of the GTEx database that had a minimum number of samples. Among the upregulated BIO‐AGEmiRNAs, *MIR4461* was the most LV‐enriched in BA‐old individuals as compared to young, with an associated SPM of 0.39 (Table S16). The expression values of *MIR4461* were significantly different in the LV as compared to other 23 tissues, except for the atrial appendage (Figure 6a). Among the downregulated BIO‐AGEmiRNAs, hsa‐mir‐490 was the most LV‐enriched in BA‐young individuals as compared to elder, having an SPM of 0.33 (Table S16). Significant differences were found when comparing its expression values with 23 other tissues except for atrial appendage and testis (Figure 6b). LV enrichment of *MIR4461* and hsa‐mir‐490 was similar across tissues over the whole range of ages (Figure [Link], [Link]a and b).

In LV from living donors, the expression of the cardiac‐enriched BIO‐AGEmiRNAs followed the trend of change with increasing BA observed in GTEx (Figure [Link], [Link]c). In addition, the preliminary correlation analysis between the levels in LV and plasma is near significance for hsa‐miR‐4461 (Spearman *R* = 0.479, *p*‐vale = 0.166) (Figure [Link], [Link]d).

Therefore, our tissue enrichment analysis identifies two cardiac‐enriched BIO‐AGEmiRNAs in the human LV, *MIR4461*, and hsa‐mir‐490, which are both present in plasma and miR‐4461 shows a tendency to reflect the levels in LV.

## 4 DISCUSSION

In this study, the transcriptional landscape of human LV aging is investigated at the whole tissue level from a biological and chronological point of view with interest in describing the contribution of miRNAs as key transcriptional regulators of genes involved in age‐induced cardiac dysfunction and as potential biomarkers of LV BA.

### 4.1 AppAge and *CDKN2A* are TA markers to assess BA in human LV

Here, we study transcriptional changes along aging comparing CA with simple (*CDKN2A* expression) and complex (AppAge) TA markers. The aim was to understand whether accounting for inter‐individual aging rate variability can uncover relevant age‐related mechanisms in human LV otherwise unseen by CA. We observed that TA markers better represent the LV transcriptome along aging and are more sensitive than CA to detect DEGs and heart‐related enriched/depleted functions. TA markers are thus more effective in unveiling organ‐specific alterations with aging. Importantly, most information uncovered by CA‐based analysis is also captured by *CDKN2A* expression or AppAge and the outcome of the single TA marker *CKDN2A* is unbiased toward its gene‐specific functions. Altogether, these findings support our working hypothesis that accounting for inter‐individual aging rate variability by TA markers improves the detection of relevant age‐related processes.

Besides its reduced sensitivity compared with AppAge, *CDKN2A* expression is set here as the BA marker because a single gene, present in LV and in many aged tissues (Idda et al., 2020), renders data susceptible of verification across studies. Since this study is carried out at the whole tissue level, the specificity of the cellular type from where *CDKN2A* is expressed is not relevant for the conclusions reached in this work.

On this basis, our research relevantly contributes to the establishment of a simple and cohort‐independent TA marker, namely *CDKN2A* expression, for the study of the molecular basis of biological aging in the human LV.

### 4.2 TA markers are biologically validated for cardiac aging

It is well established that cardiac aging entails an increase in the degree of LV fibrosis that leads to ventricular dysfunction (González et al., 2018). Here, we provide biological validation of the relation of this CA‐associated aging trait with the TA markers. At the histological level, the age‐related content of fibrosis in human LV has been analyzed only in two studies (Gazoti Debessa et al., 2001; Mendes et al., 2012). In both, comparable higher amounts of collagen deposition were observed in elder (67–87 y.o.) as compared to young persons (20–28 y.o.). Our results for CA agree with these observations, but differ in the total amount of observed fibrosis, which seems slightly higher in our case. This is likely due to the method used for quantification. Hematoxylin‐eosin staining does not specifically detect collagen fibers as Masson trichrome staining, the gold‐standard technique used to study fibrosis. Instead, it stains the whole extracellular matrix in light pink color contrasting with the dark staining of myocytes, the main cell type contributing to the tissue structure (Figure S4). Our computational quantification approach, although is pretty faithful, could account for the observed difference since all matrix fibers are detected, not just collagen. At the single‐gene level, the expression profiles of members of the TGF‐β pathway and components of the ECM confirm the trend observed at the histological level. The association of TA markers with cardiac fibrosis again highlight their increased sensitivity to account for the individual aging rate and the relevance of conducting aging studies from the biological perspective.

We, hereby, empirically demonstrate the relation of TA markers with a main cardiac aging phenotype.

### 4.3 Depleted and enriched functions in biologically and chronologically old individuals

In GSEA, the top 20 enriched GO groups by the three age markers have been mainly related to immunity, inflammation, and chemotaxis. Our study confirms previous observations such as the inflammatory response during aging (López‐Otín et al., 2013). Other enriched groups in the CA and *CDKN2A*‐based analysis are related to angiogenesis. Although there is extensive literature on impaired angiogenesis in the elderly (Obas & Vasan, 2018), our findings would agree with pathological angiogenesis related to the senescence‐associated secretory phenotype (Oubaha et al., 2016).

Commonly depleted functional processes for the three aging markers are mainly involved in mitochondrial and energy metabolism, which coincides with previous observations (López‐Otín et al., 2013). Functions related to protein synthesis are also depleted in all of them, this being in line with the well‐described alterations in the translation process during aging. Reduction in protein synthesis has been shown to range from 20% to 75% in many tissues and organisms, and translation has been reported to become selective with age, promoting the synthesis of proteins involved in repairing mechanisms and stress response (Gonskikh & Polacek, 2017).

Interestingly, our study unveils relevant cardiac‐related processes as depleted in individuals with high *CDKN2A* expression and/or high AppAge, but not in CA‐old individuals. These cardiac processes constitute up to 25% or 40% (*CDKN2A* and AppAge, respectively) of the top 20 GOs. Aside of the cardiac‐specific GOs, other CA‐undetected functions include depletion of cytoskeleton‐related processes. The appearance of cytoskeleton GOs (“Regulation of actin filament‐based movement”, “Myofibril assembly” or “Actin‐mediated cell contraction”) agrees with the mechanism of myofibril disorganization that alters cardiac contractility and shortens lifespan in Drosophila (Melkani et al., 2017). For bone and cartilage‐related functions enriched in AppAge, previous studies have reported that genes involved in cartilage development participate also in vascular calcification in the heart (Fitzpatrick et al., 2003; Trion & Van Der Laarse, 2004). Moreover, proteins involved in bone morphogenesis are necessary during heart development (van Wijk et al., 2007) and it is known that expression of fetal genes is re‐activated during aging (Dirkx et al., 2013). Therefore, both vascular calcification and emergence of fetal genes could be indicative of aging phenotypes.

In agreement with our results on fibrosis and aging, *CDKN2A*‐ and AppAge‐focused enrichment analysis yielded functional groups related to extracellular matrix organization, which is associated with fibrotic remodeling in human aged hearts (Chiao & Rabinovitch, 2015). SMAD protein‐related processes in CA could agree as well with age‐induced cardiac fibrosis (Chiao et al., 2012). The similarities between *CKDN2A*‐ and AppAge‐based analysis, as well as the great number of cardiac‐specific functions differentially depleted by *CDKN2A* and/or AppAge, further support the importance of not only considering CA but also BA in aging investigations.

Single‐cell RNA‐seq could help to determine genes and functional groups relevant for biological aging at the individual cell level. Still, whole tissue transcriptomics is highly informative as it has been demonstrated for decades. In particular, for human aging research, the high inter‐individual variability across lifespan demands using a sufficiently high number of subjects to obtain relevant results, as demonstrated in this work. Carrying out a study of the magnitude of this one by single cell RNA‐seq would be technically challenging. Our results could, however, contribute to guide insightful aging research at the single‐cell level using the recently described human heart atlas (Litviňuková et al., 2020).

In summary, our results confirm well‐known aging landmarks in human LV at the same time that BA‐based analysis demonstrates a remarkable potential to unveil novel cardiac‐specific and age‐related functional and structural impairments.

### 4.4 Identification of biological age‐related miRNAs

The important role of miRNAs in the regulation of human LV aging is shown in our study. Twenty BIO‐AGEmiRNAs are identified by *CDKN2A*‐based analysis, a 3.3% of the LV miRNome. Similar studies in other tissues reported 8% and 16.8% age‐related miRNAs (Huan et al., 2018; Simon et al., 2014). Our more stringent criteria could explain this difference. A criterion including all miRNAs with *CDKN2A*‐based differential expression would have retrieved 51 BIO‐AGEmiRNAs (8.4%), but we imposed additional conditions to also account for life‐long correlation with age in adulthood.

In the literature, a variety of miRNAs have been related to age‐induced alterations. BIO‐AGEmiRNA *MIR22HG*, downregulated with BA in our human LV data, has been previously found to be upregulated with CA in mouse heart, neonatal rat cardiomyocytes, and human atria (Gupta et al., 2016; Jazbutyte et al., 2013). miR‐22 inhibits autophagy (Gupta et al., 2016) and is essential for stress‐induced cardiac hypertrophy in mice (Huang et al., 2013), both processes involved in cardiac aging (Obas & Vasan, 2018). In the case of the downregulated miRNAs from the *MIR17HG* family (miR‐17, miR‐18a, miR‐19a, miR‐19b1, miR‐20a and, miR‐92a), some of them have been found to be downregulated in several cellular aging models (Hackl et al., 2010). Consistent with our findings, miR‐17 has been reported to inhibit senescence in mouse cardiac fibroblasts (Du et al., 2015) and miR‐19a and miR‐19b to indirectly inhibit autophagy and apoptosis in human cardiac fibroblasts. We found MIR155‐HG as upregulated. Interestingly, upregulation of miR‐155 was related to hypertrophy (Seok et al., 2014), which is tightly related to aging. Other miRNAs have been shown to play a role in the aging process in previous studies. In particular, miR‐34a has been described to be essential in promoting heart aging in mice and its expression to correlate with CA in human atria (Boon et al., 2013). However, this miRNA did not emerge in our human LV study.

Our results describe the involvement of miRNAs in the aging process and partially agree with previous reports. Most of the cited studies are based on *in vitro* or *in vivo* research in other species. Animal models, especially the small ones, have remarkable physiological differences, reduced lifespan, and lower exposure to the environment as compared to humans. All this could account for the observed discrepancies. Also, differences in the tissue of origin could play a role, like for the human atrium age‐associated mir‐34a (Boon et al., 2013). Chamber‐specific miRNA expression and response to stimuli are reported in mice (Gioffré et al., 2019; Kakimoto et al., 2016).

Our work remarks the significance of conducting aging studies from human LV samples, particularly by BA‐based analysis, and stresses the need to further investigate the role of the herein described miRNAs in human systems, like iPSC‐derived cardiac cells.

### 4.5 miRNA‐based regulation network of genes involved in human LV biological aging

In this study, predicted miRNA‐target gene regulatory networks specific of human LV biological aging are built. The network of upregulated BIO‐AGEmiRNAs contributes to the regulation of genes involved in cardiac function to a higher extent than the downregulated network (72 vs. 34 cardiac‐related genes). This agrees with our functional group enrichment results where heart‐related processes appear only as depleted during BA (Figure 3b).

Some of the mirror targets of the upregulated BIO‐AGEmiRNA network are related to cell–cell communication, a depleted functional group in the *CDKN2A*‐based GSEA (Table S12, GO:0086065). Desmosomes provide mechanical continuity in the myocardium by linking the intermediate filaments of the cytoskeleton between two adjacent myocytes and are associated with arrhythmogenic cardiomyopathies (Delmar & McKenna, 2010). *JUP*, *DSC2* and *DSP*, which encode proteins of the desmosome, are mirror targets of *MIR3916*, indicating that miR‐3916 may be responsible for desmosome destabilization during aging. Also, *MIR4435*‐*1HG* could participate in this process by downregulating *DSP*, tightly involved in conduction (Kaplan et al., 2004), an interaction that we have proven in vitro. But, lack of *MIR4435*‐*1HG* increase with BA in our pilot validation data (Figure [Link], [Link]) questions this regulation axis and asks for further investigation. Also linked to cell–cell communication and specifically related to conduction, connexin 43 (encoded by *GJA1*), the main ventricular connexin in the intercalated disks of human LV forming GAP junctions, is a mirror target of hsa‐mir‐6080. *POPDC2 *has been postulated to have a role in cell–cell communication (Soni et al., 2016), and our experimental validation shows its downregulation by hsa‐miR‐24‐2, even if in the LV tissue from living donors its expression does not significantly change with *CDKN2A* expression.

Regarding cardiac action potential, two groups are identified as downregulated in BA‐old individuals (“Cardiac muscle cell action potential” and “Regulation of cardiac muscle cell action potential”), which rely on ion channels. Indeed, from the 72 identified cardiac‐related mirror targets, 12 of them encode for ion channels and *KCNQ1*, *KCNH2*, and *ATP1A2* tend to decrease with age in living donors. Although ion channel malfunction can produce ventricular arrhythmias (Clancy & Kass, 2005), their expression has been scarcely studied in relation to aging. Only, *HCN2 *has been reported to be downregulated at the protein level in the senescent rat sinoatrial node (Huang et al., 2016) and *KCNJ11* to be mildly downregulated with aging in guinea pig hearts (Ranki et al., 2002). Here, in agreement with the bioinformatics analysis, we provide preliminary evidence of *KCNQ1*, *KCNH2*, and *ATP1A2* downregulation with BA in human LV.

Calcium handling, reported to be impaired in old individuals (Chiao & Rabinovitch, 2015), involves many genes including *ATP2A2* (*SERCA2*) and *CASQ2*, which are mirror targets of *MIR24*‐*2* and *MIR3916* (and *CASQ2* of hsa‐mir‐6080). In fact, downregulation of *SERCA2* by hsa‐mir‐24‐2 has been validated *in vitro* in this study and analyzed in samples from living donors, together with *CACNA1C*, where the later tends to decline with BA. The expression of SERCA2 is known to reduce with age in murine cardiomyocytes and human atrium (Cain et al., 1998; Chiao & Rabinovitch, 2015), but seems to remain constant in our data from living patients. Further research is needed to fully portray *SERCA2* expression changes in the aging human LV. Yet, our results suggest that these BIO‐AGEmiRNAs, could have a role in the altered intracellular calcium management in aged individuals.

Sarcomeric and cytoskeleton proteins, responsible for the physical contraction of cardiomyocytes, appear in our regulatory network as downregulated. Although many studies have connected altered sarcomeric function with age (Sessions & Engler, 2016), no studies have so far associated it with specific genes nor specific regulatory mechanisms. *TNNC1*, *TNNT2*, *ACTN2*, *ACTC1*, and *TPM1* are all mirror targets of hsa‐mir‐6080 and *MIR3916*. *TNNI1* and *MYBPC3* (a regulatory subunit of myosin) are regulated by *MIR4435*‐1HG, while *MIR24*‐*2* regulates *TTN*, *TMOD1*, *and ACTN2*. These last two interactions have been proved *in vitro* in this work, suggesting that miR‐24‐2 could be impairing the contraction process due to their participation in cytoskeleton‐related functions (Cooper, 2006; Kong & Kedes, 2006). Our results are novel in postulating that, at the transcriptional level, the expression of sarcomeric or cytoskeletal proteins, and thus cardiac contraction, could be modulated by BIO‐AGEmiRNAs.

The autonomic nervous system has a well‐known role in the modulation of heart function (Lymperopoulos et al., 2013) and specifically, α1‐adrenergic receptors (encoded by *ADRA1A*) have a protective role in cardiomyocytes (Huang et al., 2007; Wu et al., 2014). Here, we have demonstrated that *ADRA1A* expression is downregulated by hsa‐mir‐24‐2 and that its tendency is to decrease with age in living donors, suggesting that this BIO‐AGEmiRNA could take part in the age‐related cardiomyocyte damage.

In the downregulated BIO‐AGEmiRNA network (Figure 4b), there is a remarkably lower number of mirror targets than in the upregulated BIO‐AGEmiRNA network, coinciding with the observation that heart‐related functions are mainly downregulated in biologically old individuals (Figure 3).

In summary, we established a LV‐specific gene regulation network controlled by BIO‐AGEmiRNAs that could contribute to explain age‐induced LV impairments. Our study is in line with previously reported electromechanical alterations in old hearts, but it settles the need to validate every interaction of the computationally generated network and their effect *in vivo* or in human systems. This network offers an overview of miRNA‐gene regulation in the aged human LV and sets the basis for future studies aimed at designing cardiac‐specific therapies based on BIO‐AGEmiRNAs.

### 4.6 BIO‐AGEmiRNAs with potential as biomarkers of LV aging

The association of circulating blood miRNAs with age has been investigated in different studies aiming to determine age biomarkers in a non‐invasive way (Rusanova et al., 2018). However, most of the studies compare the healthy/diseased or young/old status and its association with certain miRNAs present in body fluids disregarding the miRNA tissue source (Kumar et al., 2017). This study identifies two age‐related miRNAs, *MIR4461* and hsa‐mir‐490 as highly enriched in the human LV myocardium compared with more than 20 other human tissues across aging. We find both miRNA present in plasma and the plasma levels of miR‐4461 correlate no significantly with the ones in LV. Despite a greater number of individuals would be required to properly assess this association, this result suggests that cardiac‐enriched miRNAs could inform in a minimally invasive manner about the biological age of the heart in body fluids. Specifically, given the sensitivity and specificity of RNA detection by PCR or other cutting‐edge diagnostic tools, like SHERLOCK (Gootenberg et al., 2017), cardiac‐enriched miRNAs would inform about accelerated heart aging in individuals whose plasma levels are over average values of a population chronologically contemporary, even before presenting clinical signs of heart function decline. This could allow to anticipate specific treatments to prevent any deleterious effects and improve the well‐being of many individuals whose genetics and/or environment hasten their heart aging process. Future experiments will be directed to determine their potential as heart biological aging biomarker in relation to heart function.

As a conclusion, here we propose two cardiac‐enriched miRNAs that could have a significant impact for age‐related cardiac risk prediction as biomarkers.

### 4.7 Study limitations

Research into physiological aging of the human LV is limited due to the lack of accessibility to ventricular tissue samples from non‐diseased donors. The GTEx samples analyzed in this study are obtained from tissues without macroscopic damage that undergo basic histopathological evaluation (Carithers et al., 2015). Thus, here we investigate healthy LV tissue by selecting NCD donors only. Although other comorbidities cannot be discarded, we hypothesize that the variability introduced by such variables in the LV transcriptome is accounted by the BA markers, as any other genetic and/or environmental factor, and therefore, biological aging is represented in the LV transcriptome. Our investigation consistently proves this hypothesis by showing that TA‐related analysis encompasses CA‐based outcomes and additionally uncovers other age‐related processes.

Previous transcriptomic studies on human LV samples, not necessarily related to aging, have been conducted with less than half the number of donors used here (Boheler et al., 2003; Pilbrow et al., 2012). In a previous study based on the GTEx database, age‐related transcriptomic changes in multiple tissues, including 83 LV samples, were investigated, but neither gender, health status, premortem circumstances, existence of co‐morbidities, medication, nor RNA quality‐related parameters (to mention some) were considered for the selection of donors or as analysis covariates (Yang et al., 2015). Our study selects individuals with non‐cardiac‐related deaths and includes samples from a larger number of donors (132). Although this selection does not fully exclude unknown confounding factors present in the NCD individuals, the large size of the cohort is highly likely compensating them.

Last, the use of healthy postmortem tissue as a proxy of the transcriptional changes that occur in life (LV aging in this case) could be questioned, but this is the sole mean of addressing this specific question in humans. Nevertheless, we hereby provide preliminary evidence of the preservation of the LV gene expression changes observed in the GTEx NCD cohort in a reduced group of living donors of middle to advanced age.

## 5 CONCLUSION

The adequacy for transcriptional analysis of human LV aging based on BA is shown and the potential of *CDKN2A* as a BA marker is demonstrated in comparison with another complex index measuring apparent age based on thousands of genes. Aiming at finding regulatory molecules of LV aging with potential therapeutic value, our research proposes 20 BIO‐AGEmiRNAs and their associated mirror targets. Our results point to a relevant role of miRNAs in contributing to the regulation of genes involved in human ventricular structural and functional changes during aging. The description of LV‐enriched age‐related miRNAs present in plasma constitutes a major advance in the proposal of non‐invasive biomarkers of accelerated cardiac aging.

## CONFLICT OF INTEREST

The authors declare that they have no conflict of interest.

## AUTHOR CONTRIBUTIONS

E.R‐M., L.G‐M., L.O., and E.P. conceived the study. L.O. and E.P. supervised the study. E.R‐M., L.G‐M., M.P‐Z., H.S‐B., S.S., JC.O., R.T‐P., A.C., E.G‐H., L.O., and E.P. performed experiments and analyzed the data. J.M.V‐G., PC.F‐R., J.F‐M., M.V‐S., M.M‐A., J.F.S‐B., J.A.B‐M., F.J. M‐S, A.S.V‐N., C.B‐C, M.J‐N., and J.M.V., collected the human LV and blood samples, and M.S‐R and A.O‐V processed them. C.G‐M and G.M. contributed to the histological analysis. E.D. participated in the development of the computational tool for cardiac fibrosis assessment. E.R‐M. wrote the manuscript with contributions by L.G‐M., L.O and E.P., and all the authors carefully reviewed the data and the manuscript.

## Supporting information

Supplementary Material

Figure S1

Figure S2

Figure S3

Figure S4

Figure S5

Figure S6

Table S2

Table S3

Table S4

Table S5

Table S6

Table S7

Table S8

Table S9

Table S10

Table S11

Table S12

Table S13

Table S14

Table S15

Table S16

## Data Availability

The data that support the findings of this study are openly available in dbGaP at https://www.ncbi.nlm.nih.gov/gap/, reference number phs000424.v7.p2.
