# Supplementary material for: Chronological and biological aging of the human left ventricular myocardium: Analysis of microRNAs contribution
Source: Aging Cell. 2021 Jun 6;20(7):e13383. doi: 10.1111/acel.13383 (PMC8282276; doi:10.1111/acel.13383)
Supplement: Supplementary file 1 — Supplementary Material [file ACEL-20-e13383-s002.docx]

**SUPPLEMENTAL MATERIAL**

**Supplementary methods**

**Donors and sample selection**

RNA-seq data (for both messenger RNAs and microRNAs) from a total of 318 RNA samples, of 202 male and 116 female, were obtained from LV specimens of the GTEx study Version 7 (Carithers et al. 2015). Donors’ ages ranged from 20 to 70 years old (Table S1). By using the keywords “cardiac arrest”, “acute myocardial infarction”, “cardiovascular”, “heart disease”, “cardiovascular collapse”, “cardiac failure”, “atherosclerotic cardiovascular disease”, “heart failure”, “cardiopulmonary arrest” and “cardiopulmonary failure” on the cause of death, individuals who died of cardiac-related causes (CD) were identified and removed from the study. This work is focused on the non-cardiac-related cause of death (NCD) donors. Considering the reported gender-related differences in gene expression with aging (Boheler et al. 2003) and the smaller number of female NCD donors, this study focused on the male gender. Male NCD samples were classified in decades (20-30, 31-40, 41-50, 51-60 and 61-70 y.o.) or fifths according to *CDKN2A* expression or AppAge (P20/40/60/80/100).

RNA samples of the 132 samples included in the study showed a median [Interquartile range, IQR] RIN (RNA integrity number) value of 7.50 [6.50-8.10]. Post-ischemic time, meaning the time between death and tissue preservation, had median values of 304.5 [148.25-659.75] minutes. Previous studies on the effect of postmortem interval on RNA quality suggest only minor expression changes within the first 24 hours postmortem when stored in cold conditions (Ferreira et al. 2018).

**Hierarchical clustering and age sample distribution analysis**

Normalized expression values were obtained from raw expression (read counts) of RNA-seq samples by normalization using the DESeq2 R package (version 1.10.1) (Love et al. 2014) and log2-transformation (zero values were converted to 1 prior to log2 calculation) (Table S2). For all tables shown in this study, symbol and description of each gene were retrieved using BioMart (NCBI) with assembly GRCh37.p13.

Hierarchical clustering (HC) was performed by using MeV (MultiExperiment Viewer) (Howe et al. 2010). Samples were clustered by normalized expression values of the whole transcriptome.

Sample trees were obtained by selecting Euclidean distance and average linkage clustering. EvolView was used to display the dendrogram (Subramanian et al. 2019).

AppAge*,* defined as the sum of CA and ∆-aging (calculated according to the method described in (Rhinn & Abeliovich 2017)) (Table S3).

**Quantification of myocardial fibrosis**

Histology images of hematoxylin-eosin (HE) staining corresponding to the 132 NCD donors were downloaded in .svs format from the GTEx database and transformed to .tif.

Images were analyzed with a house software tool (MATLAB R2020a, The MathWorks Inc., Natick, MA) which works via a graphical user interface (GUI). After loading and displaying the .tif image to analyze, the custom software performs automatic masks extraction for the tissue (myocytes) or the extracellular matrix and quantifies the percentage of the fibrotic tissue. This tool is part of a software for histological image analysis that is under development in an article in preparation (Pérez-Zabalza et al. 2021).

We generated the masks obtaining first the representative RGB color of each component, the tissue and the extracellular matrix, by exploring interactively the pixel color values through the image. Once defined, the tissue and the matrix masks were identified as those pixels whose absolute RGB value minus the representative RGB value were less than 20 and 10, respectively. A black mask was created for such pixels and the rest of values were included in a white mask. For visualization purposes, the tissue mask was depicted as red, the extracellular matrix mask as blue and the merge as black (Figure S4). RGB values were adjusted when necessary to eliminate merge.

The percentage of cardiac fibrosis was calculated by dividing the number of black pixels of the extracellular matrix mask over the sum of white pixels of both masks.

**Differential gene expression and whole transcriptome gene set enrichment analysis**

GSEA software version 3.0 software (Subramanian et al. 2005; Mootha et al. 2003) was used to define functional groups (Gene Ontology of biological processes, from MSigDB v6.2) of genes enriched or depleted in individuals having high AppAge values, high CDKN2A expression and CA-old. Settings used in the GSEA are the following: group size was limited from 3 to 500 genes and the number of permutations was set to 1000. GSEA pre-ranked option was used to evaluate the 54320 genes annotated in the RNA-seq whole transcriptome analysis. The differential expression values (LogRatio) for each gene were obtained by using DESeq2 R package in donors with high/low AppAge (P100 and P20), high/low *CDKN2A* expression (P100 and P20) or old/young CA (61-70 and 20-30 y.o.) (Table S4).

Venn diagrams were created using Venny 2.1.0 (<https://bioinfogp.cnb.csic.es/tools/venny/index.html>) (Oliveros 2007).

**miRNA expression profile calculation**

To obtain gene expression profiles along aging, the RNA-seq samples were normalized using the quantiles (*normalizeQuantiles*) method of the R *limma* package (Ritchie et al. 2015) and the values were log2-transformed as indicated above. For each gene, the average of the normalized log2-expression in each interval (*CDKN2A* expression or AppAge quintile) was computed (interval average). A baseline expression was calculated for each gene as the average of the five interval averages. The ratio of gene expression for each *CDKN2A* or AppAge quintile was computed by subtracting the baseline value (Table S7).

**Identification of age-related miRNAs**

The human LV miRNome consisted of 1314 annotated miRNAs, although miRNAs with no reads in any of the studied individuals were removed, thus leaving 608 miRNAs and only those miRNAs with RNA-seq normalized expression values (Table S2) in at least 30% of individuals were included in the analysis, rendering a total of 93 miRNAs for further analysis (Table S5). Since aging is a continuous process in time, BIO-AGEmiRNAs were identified not only as those being differentially expressed (DEG) between the highest/lowest *CDKN2A* expressing groups (Table S4), but also showing significant Spearman correlation with *CDKN2A* expression (Table S6).

**Selection of mirror targets for BIO-AGEmiRNAs**

The miRWalk database (<http://zmf.umm.uni-heidelberg.de/apps/zmf/mirwalk2/index.html>) (Sticht et al. 2018) was used to identify BIO-AGEmiRNA targets. The predicted targets were filtered to mirror the expression profile of their associated BIO-AGEmiRNA. Specifically, the expression patterns obtained above (Table S7) were used to calculate the slopes between consecutive *CDKN2A* quintiles. For steep BIO-AGEmiRNA slopes (above 0.15 or below -0.15), predicted target genes were required to present slopes of opposite and, particularly for very steep BIO-AGEmiRNA slopes (above 0.5 or below -0.5), corresponding target slopes were required to be of at least half its magnitude. For flat BIO-AGEmiRNA slopes (absolute value below 0.15), target slopes were required not to exceed 0.25 in absolute value. In addition, these identified targets were further assessed for correlation with *CDKN2A* expression, this being of opposite sign to the corresponding BIO-AGEmiRNA, and significant differential expression. The predicted target genes meeting all the above described criteria were defined as mirror targets.

**Establishment of LV-specific BIO-AGEmiRNA downstream gene regulation network**

Cardiac-related GOs were selected from the full GO list (Table S11) by including those with any of the following keywords in their group name: “heart”, “cardiac”, “ion”, “cytoskeleton”, “action potential”, “actin” or “catecholamine”. All the mirror targets were functionally annotated into GO groups. BIO-AGEmiRNAs and their associated mirror targets present in cardiac GOs were used to construct the network. For ease and simplicity, cardiac GOs were grouped into five functional categories with direct contribution to “Action potential”, “Heart rate”, “Cardiac contraction”, “Cardiac conduction” and “Autonomic Nervous System” divided in turn into sections (Table S8). It should be noted that some genes belonged to several cardiac GOs and could thus be represented in more than one functional category.

**Luciferase reporter assay**

The vector used, pmirGLO-P2A-3fLAG-MCS, was a modification of pmirGLO (Promega). Briefly, downstream of *Firefly* luciferase, the autocatalytic peptide P2A, the tag 3xFlag and a MCS were inserted. The 5’UTR, coding region and 3’UTR were cloned form most of genes in the MCS. For DSP, only the coding region was cloned. Regions of each gene cloned and primers used for cloning are included in Table S9.

HEK-293 cells were transfected with plasmid (1ng/ul) and miRNA mimic (1uM) (Ribbox M-00202) or negative control N1 (Riboxx, K-01000) with Lipofectamine 2000 (Invitrogen, 11668027). After 24h, the luciferase assay was conducted with Dual-Glo Luciferase Assay Kit (Promega, E2940) according to the manufacturer’s instruction.

**Gene expression analysis on human LV samples from living donors.**

Blood and transmural tissue biopsies from the LV anterior wall (near the base) of patients undergoing coronary artery surgery were collected at University Hospital Miguel Servet (Zaragoza, Spain) and Hospital Universitario Virgen de la Victoria (Málaga, Spain) following procedures described previously (Oliván-Viguera et al. 2020). The study conforms to the principles outlined in the Declaration of Helsinki and was approved by the local Ethics Committees (CEICA, reference number PI17/0023 and CEI from 28/09/2017). All biopsies were taken from nonischemic ventricular myocardium of patients with absent LV hypertrophy, with normal LV systolic function and without ventricular remodelling (LV ejection fraction above 50%). A total of 33 LV and 14 blood samples were obtained in age ranges from 50 to 83 years old with median value 68 and IQR [60-73] (Table S14). Plasma was isolated right after blood extraction and stored at -80ºC. LV samples were immediately processed and snap frozen in liquid nitrogen. Tissue RNA was extracted using the AllPrep DNA/RNA/miRNA Universal Kit (Qiagen) and 200 ng of total RNA was retrotranscribed with qScript XLT cDNA SuperMix (Quanta Biosciences). Plasma RNA was extracted with miRNeasy Serum/Plasma Kit (Qiagen). miRNA was retrotranscribed from 50 ng of total LV RNA or 3.5 µl of plasma RNA using the qScript microRNA cDNA Synthesis Kit (Quanta Biosciences). Real time PCR was carried out with Power SYBR Green PCR Master Mix (Thermo Fisher Scientific) using the oligonucleotides listed in Table 15 in a Viia7 instrument (Thermo Fisher Scientific). mRNA expression was normalized to *YHWAZ*, *POLR2A* and *IPO8* reference genes (Molina et al. 2018). miRNA expression was normalized to *RNU6* and *cel-miR-39-5p* (spiked in plasma samples before RNA isolation) in LV or plasma, respectively. After the PCR run, individuals were classified for *CDKN2A* expression (based on ∆Ct) and grouped in fifths. Relative gene expression was calculated according to the 2^-∆∆Ct^ method between the first (low *CKDN2A*) and fifth (high *CDKN2A*) quintiles using as reference sample the individual with the lowest ∆Ct value for each gene. Non-relative gene expression was calculated as 2^-∆Ct^. For comparison purposes, a CA-matched subset of GTEx donors was selected, individuals were categorized by *CDKN2A* expression (RPKM) and grouped into fifths. Relative expression between low (P20) and high (P100) *CDKN2A*-expressing individuals of each gene was calculated in this case by dividing the RPKM value of each sample by the reference sample (the one with lowest RPKM value).

**Tissue specificity assessment**

SPM (Pan et al. 2013) of BIO-AGEmiRNA was calculated using the reads per kilobase per million mapped reads (RPKM) values of all individuals from GTEx V7 ([GTEx_Analysis_2016-01-15_v7_RNASeQCv1.1.8_gene_reads.gct.gz](https://storage.googleapis.com/gtex_analysis_v7/rna_seq_data/GTEx_Analysis_2016-01-15_v7_RNASeQCv1.1.8_gene_reads.gct.gz)). The following tissues were excluded from the analysis: transformed cells (EBV-transformed lymphocytes and transformed fibroblasts), female tissues (ovary, uterus, vagina, cervix and fallopian tubes) and whole blood, to include in the detection LV-specific BIO-AGEmiRNAs secreted to the bloodstream. 44 tissues, including LV, remained. Since not all of them had samples for all individuals, only those with available samples for at least 30% of the NCD individuals were included in the analysis, rendering a total of 26 tissues in the P20 group (young BA) and 25 tissues in the P100 group (old BA). When all individuals were analyzed, the analysis rendered 27 tissues. SPM values calculated for LV (Table S16) can range from 0 to 1, with 1 indicating that a gene is specifically expressed in LV. Statistical differences in the expression levels of BIO-AGEmiRNAs between LV and each of the other analyzed tissues were determined by Mann-Whitney tests.

**Supplementary references**

Boheler KR, Volkova M, Morrell C, Garg R, Zhu Y, Margulies K, Seymour A-M & Lakatta EG (2003) Sex- and age-dependent human transcriptome variability: implications for chronic heart failure. *Proceedings of the National Academy of Sciences of the United States of America* 100, 2754–9. https://doi.org/10.1073/pnas.0436564100.

Carithers LJ, Ardlie K, Barcus M, Branton PA, Britton A, Buia SA, Compton CC, DeLuca DS, Peter-Demchok J, Gelfand ET, Guan P, Korzeniewski GE, Lockhart NC, Rabiner CA, Rao AK, Robinson KL, Roche N V., Sawyer SJ, Segrè A V., Shive CE, Smith AM, Sobin LH, Undale AH, Valentino KM, Vaught J, Young TR & Moore HM (2015) A Novel Approach to High-Quality Postmortem Tissue Procurement: The GTEx Project. *Biopreservation and Biobanking* 13, 311–319. https://doi.org/10.1089/bio.2015.0032.

Ferreira PG, Muñoz-Aguirre M, Reverter F, Sá Godinho CP, Sousa A, Amadoz A, Sodaei R, Hidalgo MR, Pervouchine D, Carbonell-Caballero J, Nurtdinov R, Breschi A, Amador R, Oliveira P, Çubuk C, Curado J, Aguet F, Oliveira C, Dopazo J, Sammeth M, Ardlie KG & Guigó R (2018) The effects of death and post-mortem cold ischemia on human tissue transcriptomes. *Nature Communications* 9. https://doi.org/10.1038/s41467-017-02772-x.

Howe E, Holton K, Nair S, Schlauch D, Sinha R & Quackenbush J (2010) MeV: MultiExperiment viewer. In *Biomedical Informatics for Cancer Research*. Springer US, pp.267–277. https://doi.org/10.1007/978-1-4419-5714-6_15.

Love MI, Huber W & Anders S (2014) Moderated estimation of fold change and dispersion for RNA-seq data with DESeq2. *Genome Biology* 15, 550. https://doi.org/10.1186/s13059-014-0550-8.

Molina CE, Jacquet E, Ponien P, Muñoz-Guijosa C, Baczkó I, Maier LS, Donzeau-Gouge P, Dobrev D, Fischmeister R & Garnier A (2018) Identification of optimal reference genes for transcriptomic analyses in normal and diseased human heart. *Cardiovascular Research* 114, 247–258. https://doi.org/10.1093/cvr/cvx182.

Mootha VK, Lindgren CM, Eriksson K-F, Subramanian A, Sihag S, Lehar J, Puigserver P, Carlsson E, Ridderstråle M, Laurila E, Houstis N, Daly MJ, Patterson N, Mesirov JP, Golub TR, Tamayo P, Spiegelman B, Lander ES, Hirschhorn JN, Altshuler D & Groop LC (2003) PGC-1alpha-responsive genes involved in oxidative phosphorylation are coordinately downregulated in human diabetes. *Nature genetics* 34, 267–273. https://doi.org/10.1038/ng1180.

Oliván-Viguera A, Pérez-Zabalza M, García-Mendívil L, Mountris KA, Orós-Rodrigo S, Ramos-Marquès E, Vallejo-Gil JM, Fresneda-Roldán PC, Fañanás-Mastral J, Vázquez-Sancho M, Matamala-Adell M, Sorribas-Berjón F, Bellido-Morales JA, Mancebón-Sierra FJ, Vaca-Núñez AS, Ballester-Cuenca C, Marigil MÁ, Pastor C, Ordovás L, Köhler R, Diez E & Pueyo E (2020) Minimally invasive system to reliably characterize ventricular electrophysiology from living donors. *Scientific Reports* 10. https://doi.org/10.1038/s41598-020-77076-0.

Oliveros JC (2007) Venny. An interactive tool for comparing lists with Venn’s diagrams.

Pan J-B, Hu S-C, Shi D, Cai M-C, Li Y-B, Zou Q & Ji Z-L (2013) PaGenBase: A Pattern Gene Database for the Global and Dynamic Understanding of Gene Function A. Palsson, ed. *PLoS ONE* 8, e80747. https://doi.org/10.1371/journal.pone.0080747.

Pérez-Zabalza M, García-Mendívil L, Mountris K, Oliván-Viguera A, Vallejo-Gil JM, Fresneda-Roldán PC, Fañanás-Mastral J, Vázquez-Sancho M, Matamala-Adell M, Sorribas-Berjón F, Bellido-Morales JA, Mancebón-Sierra FJ, Vaca-Núñez AS, Ballester-Cuenca C, Ordovás L & Pueyo E (2021) Age-Associated Changes in Myocardial Fibrosis Amount and Distribution Quantified from Nonlinear Optical Microscopy Images. *CinC2021*.

Rhinn H & Abeliovich A (2017) Differential Aging Analysis in Human Cerebral Cortex Identifies Variants in TMEM106B and GRN that Regulate Aging Phenotypes. *Cell Systems* 4, 404-415.e5. https://doi.org/10.1016/j.cels.2017.02.009.

Ritchie ME, Phipson B, Wu D, Hu Y, Law CW, Shi W & Smyth GK (2015) Limma powers differential expression analyses for RNA-sequencing and microarray studies. *Nucleic Acids Research* 43, e47. https://doi.org/10.1093/nar/gkv007.

Sticht C, De La Torre C, Parveen A & Gretz N (2018) Mirwalk: An online resource for prediction of microrna binding sites. *PLoS ONE* 13. https://doi.org/10.1371/journal.pone.0206239.

Subramanian a., Tamayo P, Mootha VK, Mukherjee S, Ebert BL, Gillette M a., Paulovich a., Pomeroy SL, Golub TR, Lander ES & Mesirov JP (2005) Gene set enrichment analysis: A knowledge-based approach for interpreting genome-wide expression profiles. *Proceedings of the National Academy of Sciences* 102, 15545–15550. https://doi.org/10.1073/pnas.0506580102.

Subramanian B, Gao S, Lercher MJ, Hu S & Chen WH (2019) Evolview v3: A webserver for visualization, annotation, and management of phylogenetic trees. *Nucleic Acids Research* 47, W270–W275. https://doi.org/10.1093/nar/gkz357.

**SUPPLEMENTARY FIGURES LEGENDS**

**Figure S1. Representation of Apparent Age.**

Age-related genes are identified by Pearson correlation analysis (FDR<0.05). Delta-aging (∆) is calculated for each gene by linear regression as the difference between the actual gene expression value and the expected (regressed) one. The image represents an individual whose age-related gene expression corresponds to and older apparent age than the actual one (red) and the opposite (green). The aging rate of the individual (∆-aging) is computed as the aggregate of ∆ values of all the age-related genes. Then, the apparent age (AppAge) of the individual is calculated as the sum of CA and the aging-rate factor.

**Figure S2. Differentially expressed genes according to the three aging parameters.**

Venn diagrams show the number upregulated (**a**) or downregulated (**b**) differentially expressed genes according to CA, *CDKN2A* and AppAge. “Upregulated” and “downregulated” indicate old > young and old < young, respectively.

**Figure S3. Identification of BIO-AGEmiRNAs mirror targets.**

**a)** Representative image of the procedure used to mirror targets identification. Upregulated miRNA expression pattern (green) is used to outline an exact mirror pattern (black). The expression profiles of all predicted targets are shown in blue, while those fitting the mirror pattern (mirror genes) are shown in red.

**b)** Idem as in (a) for downregulated miRNA (red) and upregulated mirror targets (green)

**Figure S4. Quantification of fibrotic tissue in samples of the GTEx study.**

Representative hematoxylin-eosin images of individuals with low and high content of fibrosis in LV (bottom). The corresponding masks of each image depict myocytes in red and extracellular matrix in blue (top). Scale bars are 300 µm.

**Figure S5. Gene expression of BIO-AGEmiRNA and mirror targets in LV of living donors.**

Relative gene expression (fold change, 2^-∆∆Ct^) in LV samples from living (top row) and GTEx (bottom row) donors with low (grey dots) and high (black dots) *CDKN2A* expression. The chronological age (age) of the selected individuals is also represented (Mann-Whitney test **, p<0.01; ***, p<0.001).

**Figure S6.** **Assessment of BIO-AGEmiRNA tissue specificity.**

Box plots show the expression levels (RPKM) of the two BIO-AGEmiRNAs with the highest LV specificity: MIR4461 **(a)** and hsa-mir-490 **(b)** in all NCD individuals. Median RPKM values are shown in red lines, p-values from Mann-Whitney tests comparing LV with each of the other tissues are indicated. **(c)** Relative gene expression (fold change, 2-∆∆Ct) in LV samples from living donors with low (grey dots) and high (black dots) CDKN2A expression. The chronological age (age) of the selected individuals is also represented (Mann-Whitney test *, p<0.05). **(d)** Spearman correlation analysis of the levels (2^-∆Ct^) of mature miR-490-5p, miR-490-3p and miR-4461 in plasma and LV paired samples.

**SUPPLEMENTAL TABLES AND TABLE LEGENDS**

**Table S1. Left ventricle samples from GTEx consortium.** Donors whose death was unrelated to cardiovascular causes were distributed in age ranges from date of birth.

| **Number of donors** | | |
| --- | --- | --- |
| **Total** | | **202** |
| Non-cardiac death (NCD) | | 132 |
| NCD age range | 20-30 | 12 |
|  | 31-40 | 13 |
|  | 41-50 | 25 |
|  | 51-60 | 50 |
|  | 61-70 | 32 |

**Table S2. DESeq2 normalized matrix.**

Matrix including the normalized expression values for each gene in all analyzed donors. Donors are identified with the GTEx codification followed by their chronological age (_XX).

**Table S3. Aging parameters.**

Chronological age, AppAge and CDKN2A values of each individual.

**Table S4. Differential gene expression in AppAge-, *CDKN2A*- and CA-classified individuals.**

Log ratio between samples classified in P100 (high AppAge) and P20 (low AppAge) groups, P100 (high *CDKN2A* expression) and P20 (low *CDKN2A* expression) groups or samples classified as Old (61-70 years old) and Young (20-30 years old). Statistical significance of the Log ratio value is shown as FDR.

**Table S5. Expressed miRNAs in the LV.**

**Table S6. Correlation between gene expression and AppAge, *CDKN2A* and CA.**

Rho and FDR of Spearman correlation test is indicated for each gene in relation to BA or CA.

**Table S7. Gene expression patterns along *CDKN2A* expression.**

Matrix used to build miRNA clusters and to identify mirror targets. Columns P20, P40, P60, P80 and P100 correspond to the average expression of each gene in the 1^st^, 2^nd^, 3^rd^, 4^th^ and 5^th^ quintiles of *CDKN2A* expression values.

**Table S8. Classification of GO groups into sections and functional categories.**

**Table S9. Gene regions cloned into pmirGLO vector and cloning oligonucleotides.**

**Table S10. Amount of cardiac fibrosis.**

Percentage of fibrosis for each individual of the NCD cohort.

**Table S11. Full outcome of GSEA in high AppAge, high *CDKN2A* and CA-old individuals.**

Enriched and depleted functional GO groups are listed giving the name of group, the number of genes in it, the enrichment score (ES), the normalized enrichment score (NES), nominative p-value (NOM p-val), false discovery rate q-value (FDR q-val), rank at maximum (RANK AT MAX) and leading edge.

**Table S12. Top 20 depleted and enriched functions in high AppAge, high *CDKN2A* and CA-old individuals.**

Top 20 depleted and enriched functional GO groups in high AppAge, high *CDKN2A* and CA-old individuals included in the wider groups.

**Table S13. Annotation of mirror targets of each BIO-AGEmiRNA into cardiac GOs.**

**Table S14. Tissue biopsies from living donors.**

**Table S15. Gene expression assays.**

**Table S16. SPM values of each BIO-AGEmiRNA.**
