# Supplementary figures and images for: Chronological and biological aging of the human left ventricular myocardium: Analysis of microRNAs contribution
Source: Aging Cell. 2021 Jun 6;20(7):e13383. doi: 10.1111/acel.13383 (PMC8282276; doi:10.1111/acel.13383)

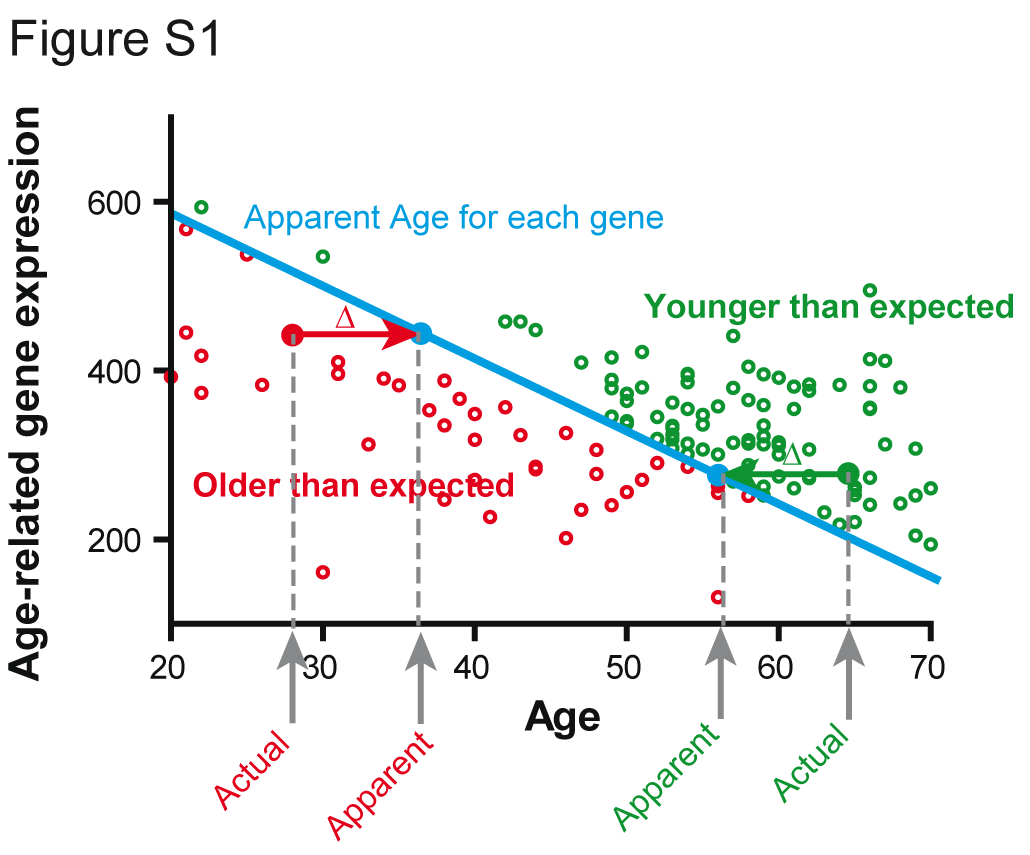

Supplement: Supplementary file 2 — Figure S1 [file ACEL-20-e13383-s005.tif]

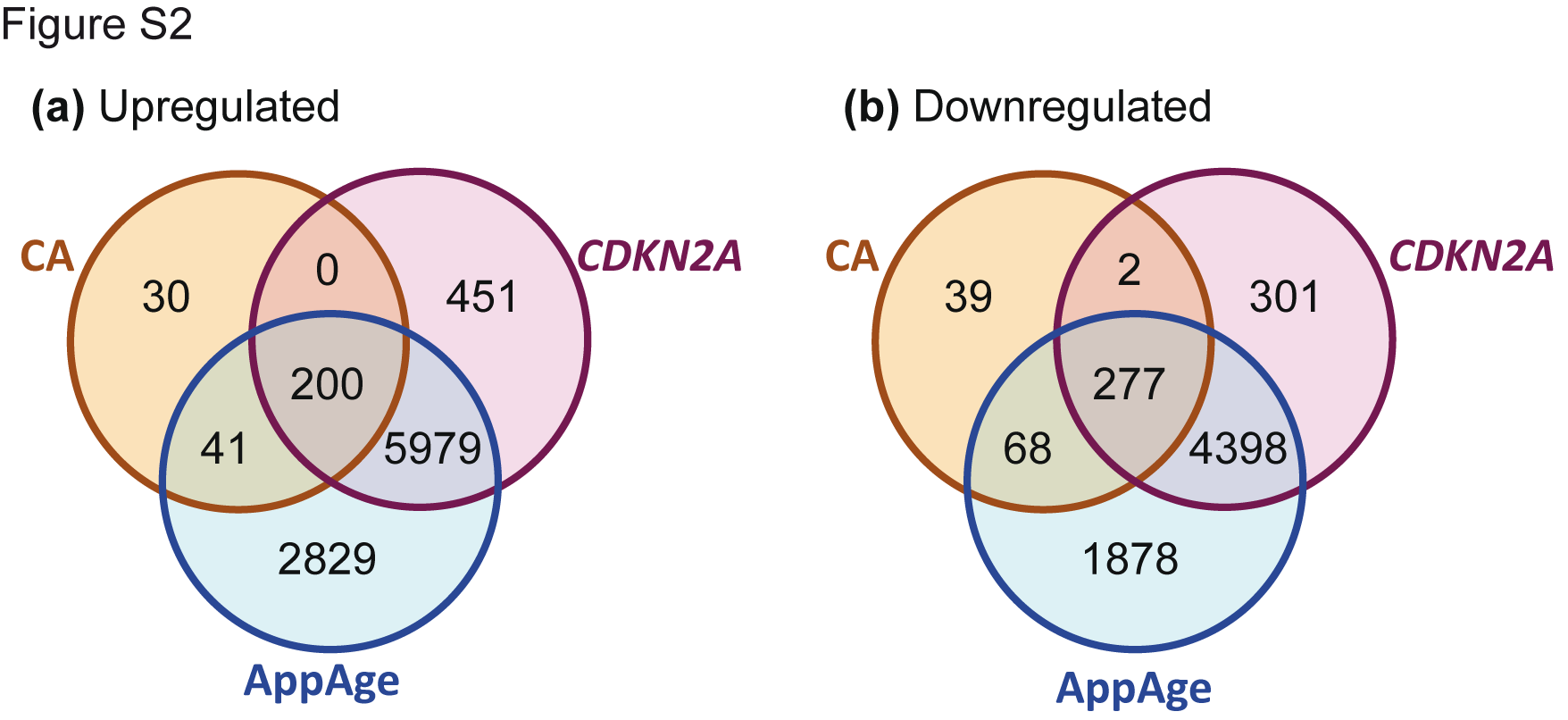

Supplement: Supplementary file 3 — Figure S2 [file ACEL-20-e13383-s016.tif]

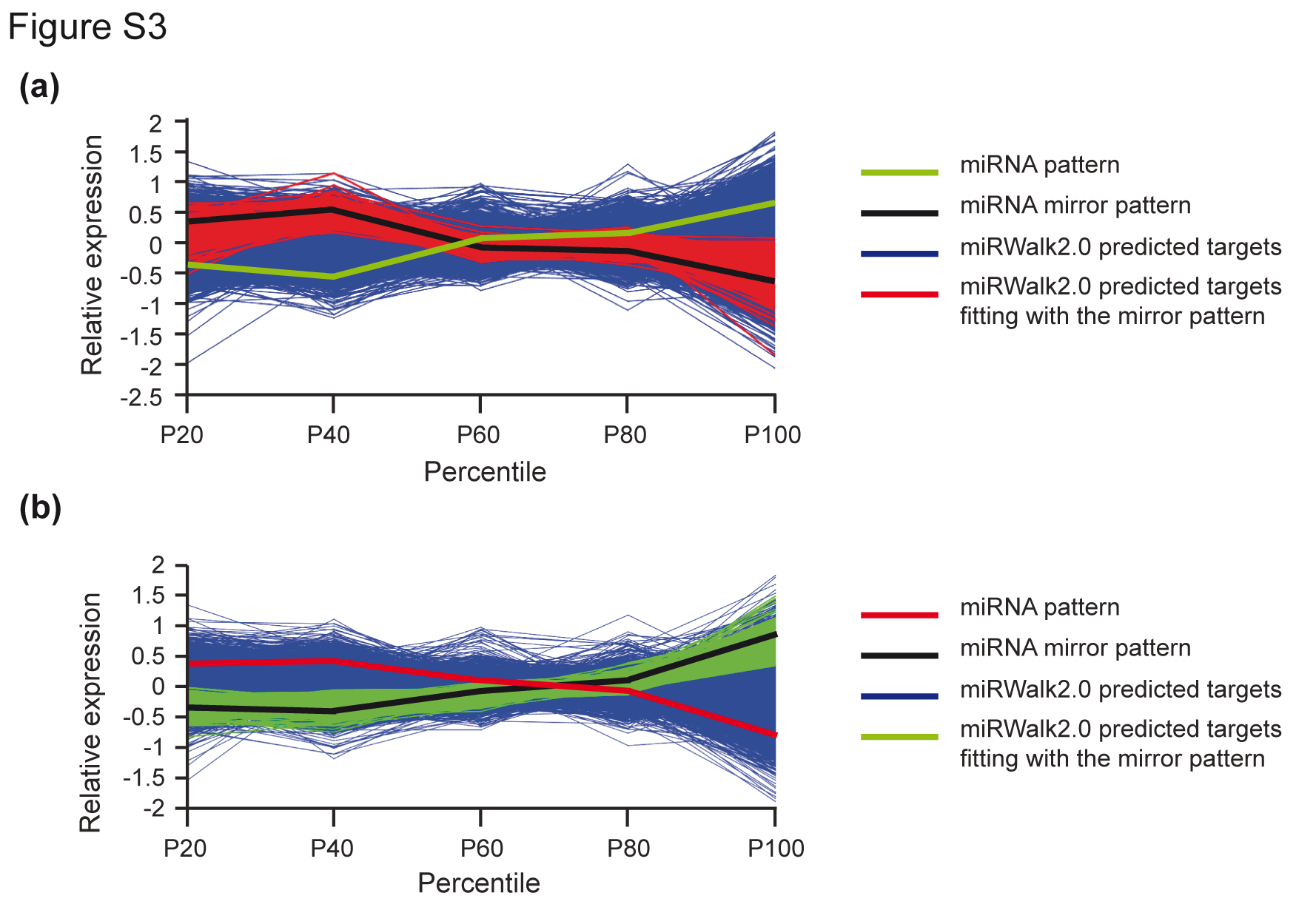

Supplement: Supplementary file 4 — Figure S3 [file ACEL-20-e13383-s015.tif]

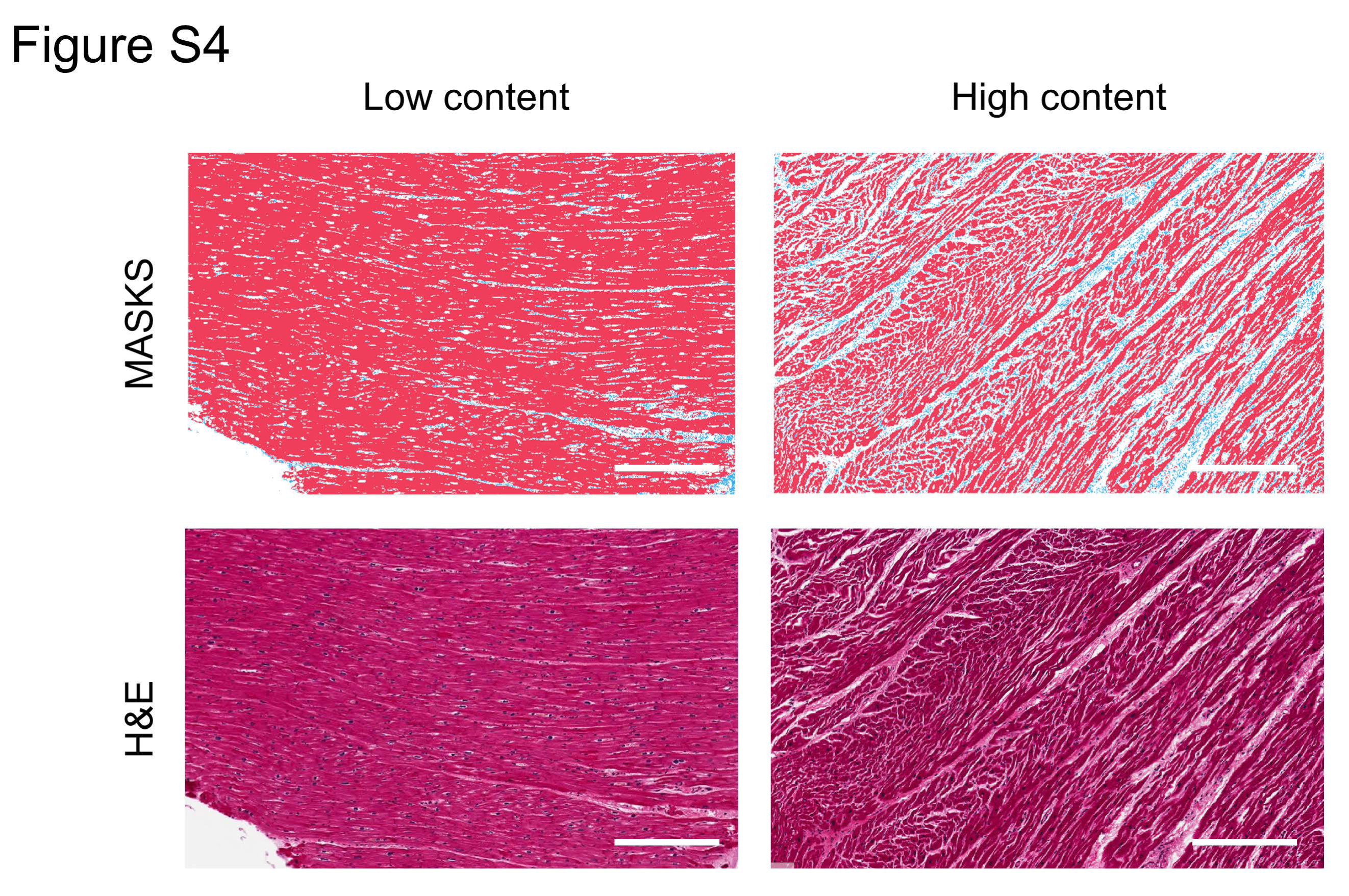

Supplement: Supplementary file 5 — Figure S4 [file ACEL-20-e13383-s013.tif]

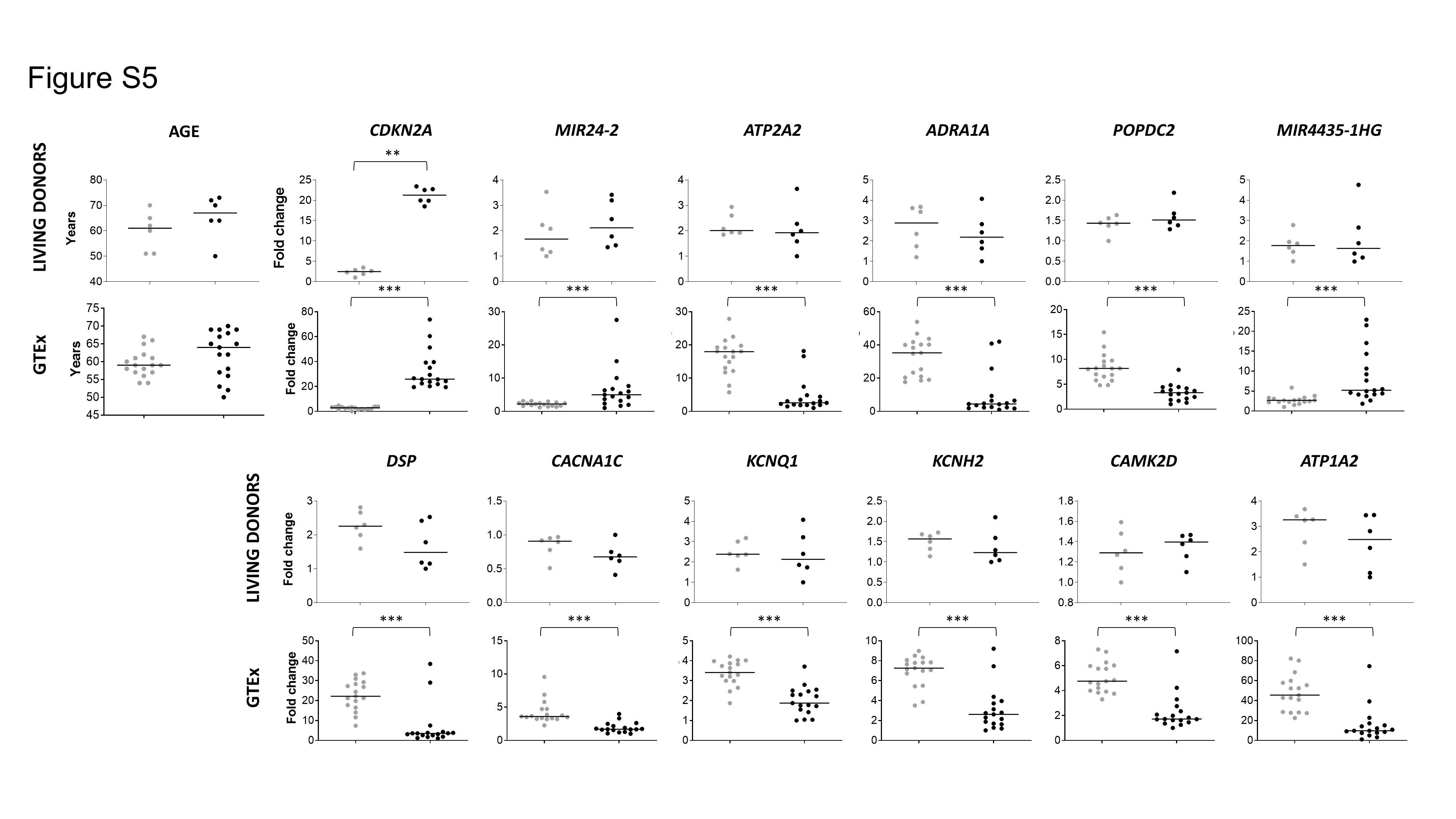

Supplement: Supplementary file 6 — Figure S5 [file ACEL-20-e13383-s022.tif]

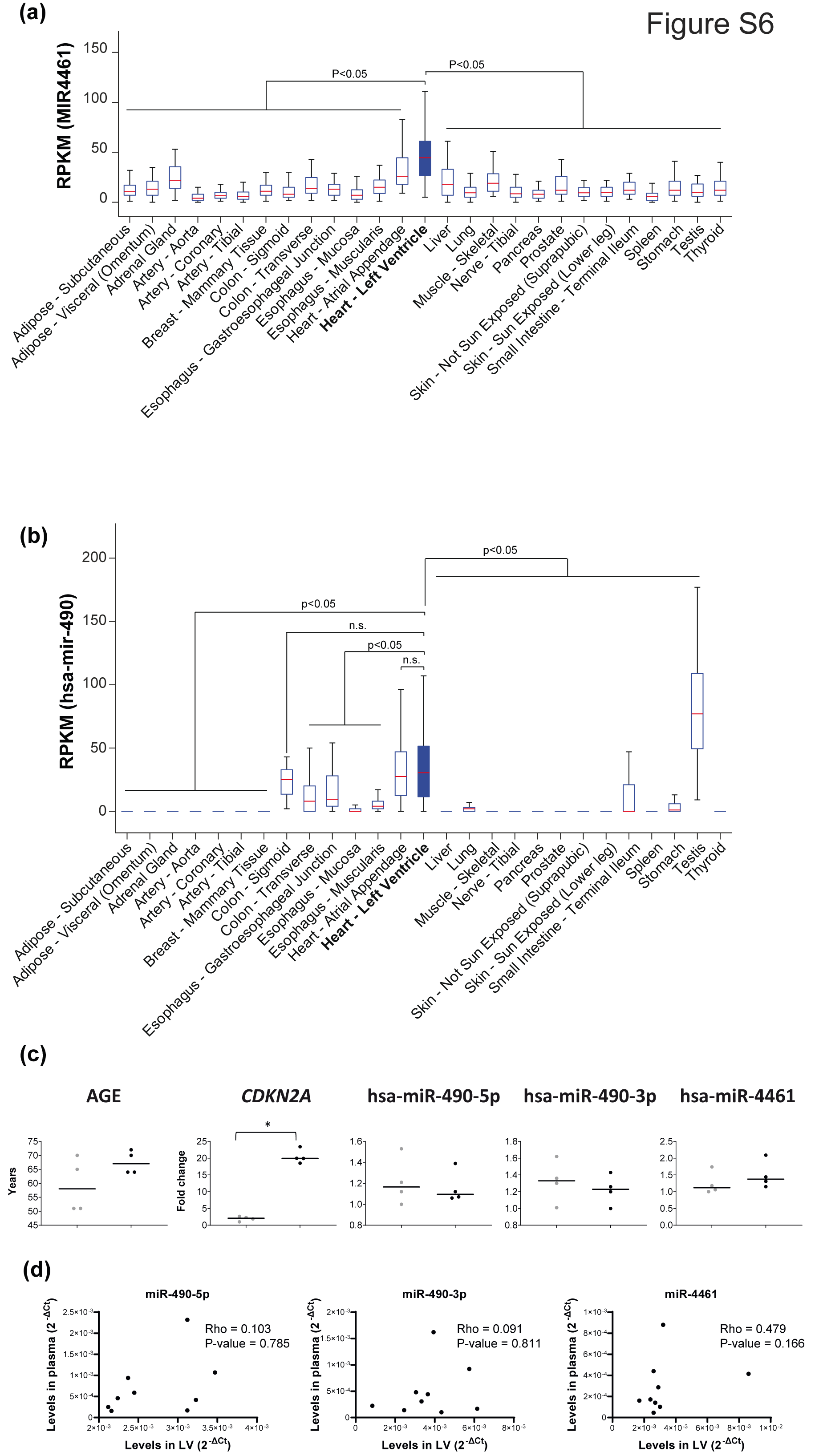

Supplement: Supplementary file 7 — Figure S6 [file ACEL-20-e13383-s008.tif]
